# New Ophiobolin Derivatives from the Marine Fungus *Aspergillus flocculosus* and Their Cytotoxicities against Cancer Cells

**DOI:** 10.3390/md17060346

**Published:** 2019-06-11

**Authors:** Byeoung-Kyu Choi, Phan Thi Hoai Trinh, Hwa-Sun Lee, Byeong-Woo Choi, Jong Soon Kang, Ngo Thi Duy Ngoc, Tran Thi Thanh Van, Hee Jae Shin

**Affiliations:** 1 Department of Marine Biotechnology, University of Science and Technology (UST), 217 Gajungro, Yuseong-gu, Daejeon 34113, Korea; choibk4404@kiost.ac; 2 Marine Natural Products Chemistry Laboratory, Korea Institute of Ocean Science and Technology, 385 Haeyang-ro, Yeongdo-gu, Busan 49111, Korea; hwasunlee@kiost.ac (H.-S.L.); choibw0924@gmail.com (B.-W.C.); 3 Nhatrang Institute of Technology Research and Application, Vietnam Academy of Science and Technology, 02 Hung Vuong, Nha Trang 650000, Vietnam; phanhoaitrinh84@gmail.com (P.T.H.T.); ngoduyngoc@nitra.vast.vn (N.T.D.N.); tranthanhvan@nitra.vast.vn (T.T.T.V.); 4 Graduate University of Science and Technology, Vietnam Academy of Science and Technology, 18 Hoang Quoc Viet, Cau Giay, Ha Noi 100000, Vietnam; 5 Laboratory Animal Resource Center, Korea Research Institute of Bioscience and Biotechnology, 30 Yeongudanjiro, Cheongju 28116, Korea; kanjon@kribb.re.kr

**Keywords:** ophiobolins, marine fungus, *Aspergillus flocculosus*, anti-proliferation

## Abstract

Five new sesterterpenes, 14,15-dehydro-6-*epi*-ophiobolin K (**1**), 14,15-dehydro- ophiobolin K (**2**), 14,15-dehydro-6-*epi*-ophiobolin G (**3**), 14,15-dehydro-ophiobolin G (**4**) and 14,15-dehydro-(*Z*)-14-ophiobolin G (**5**), together with four known ophiobolins (**6**–**9**) were isolated from the marine fungus *Aspergillus flocculosus* derived from the seaweed *Padina* sp. collected in Vietnam. The five new ophiobolins were first isolated as ophiobolin derivatives consisting of a fully unsaturated side chain. Their structures were elucidated via spectroscopic methods including 1D, 2D NMR and HR-ESIMS. The absolute configurations were determined by the comparison of chemical shifts and optical rotation values with those of known ophiobolins. All compounds (**1**–**9**) were then evaluated for their cytotoxicity against six cancer cell lines, HCT-15, NUGC-3, NCI-H23, ACHN, PC-3 and MDA-MB-231. All the compounds showed potent cytotoxicity with GI_50_ values ranging from 0.14 to 2.01 μM.

## 1. Introduction

The marine environment is an enormous reservoir of novel sources of biologically active metabolites, many of which display unique structural skeletons that can be used as lead structures for the development of new drugs [1,2]. To adapt and live in an environment that is significantly different from terrestrial organisms, marine organisms frequently produce structurally unique chemical compounds [3,4]. Specifically, secondary metabolites from marine microorganisms are recognized as a novel chemical source for drug discovery and development. Among marine-derived microbes, marine fungi produce a wide range of promising biologically active compounds [5]. Numerous novel compounds from marine fungi have displayed a wide range of bioactivities such as antiviral, antibacterial, anticancer, antiplasmodial and anti-inflammatory [6,7,8]. In the marine context, *Aspergillus* and *Penicillium* represent the best studied fungal genera as depicted in marine contexts [9,10]. The genus *Aspergillus* is known as a major contributor of pharmacologically bioactive compounds, including anticancer asperazine, antibacterial varixanthone and antifungal amphotericin B [11,12].

**Figure 1:**
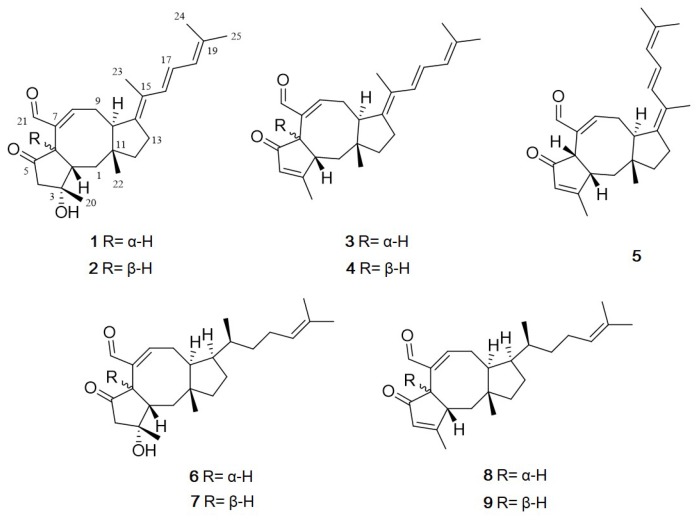
Structures of **1**–**9** isolated from *Aspergillus flocculosus*.

Ophiobolins are a group of sesterpenoids with an unusual tricyclic 5-8-5 ring system. They show a broad range of inhibitory activities against nematodes, fungi, bacteria and cytotoxic activity against cancer cells [13,14]. They are produced by the fungal genus *Bipolaris*, *Aspergillus*, *Sarocladium* and *Drechslera* [15]. The first ophiobolin, ophiobolin A, was isolated from *Biolaris* spp. and displays inhibitory activity against calmodulin-activated cyclic nucleotide phosphodiesterase [16]. These findings made the compound a useful calmodulin probe for research purposes and implied an application in anti–cancer therapy [17]. Interestingly, more than half of the 49 ophiobolins identified between 1999 and 2016 exhibit cytotoxic activities against human cancer cell lines [18]. Although their biological properties have been well exploited in recent years, their structure-activity relationship remains unestablished [18]. Consequently, this study focused on the discovery of bioactive natural products from marine fungi. During our ongoing investigation for new bioactive compounds from marine microorganisms, the fungal 168ST-16.1 strain was isolated from the seaweed *Padina* sp. collected at Da Nang, Vietnam, and, based on its 28S rRNA gene sequence, it was identified as *Aspergillus flocculosus*. Subsequent chemical investigations on an EtOAc extract of the fungal culture broth using reversed-phase HPLC led to the isolation of the five new ophiobolins, named, 14,15-dehydro-6-*epi*-ophiobolins K and G (**1** and **3**), 14,15-dehydro-ophiobolins K and G (**2** and **4**) and 14,15-dehydro-(*Z*)-14-ophiobolin G (**5**), together with four known ophiobolins, 6-*epi*-ophiobolins C and N (**6** and **8**) and ophiobolins C and N (**7** and **9**) [19,20,21] (Figure 1). Herein, details of the structure elucidation and biological activity of these compounds are described.

## 2. Results and Discussion

**Figure 2:**
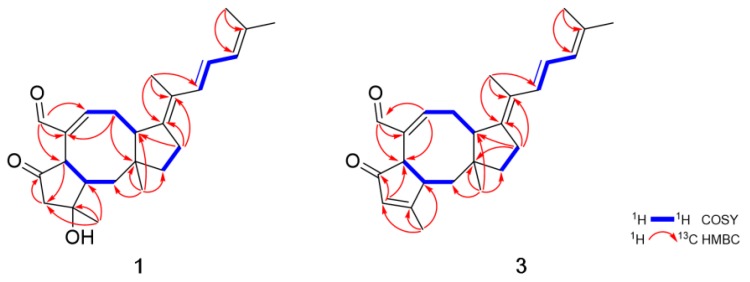
Key COSY and HMBC correlations of **1** and **3**.

Compound **1** was obtained as an amorphous powder. The molecular formula of **1** was determined to be C_25_H_34_O_3_ based on HRESIMS. The ^1^H NMR spectroscopic data of **1** displayed resonances for an aldehyde proton (*δ*_H_ 9.23), four olefinic protons (*δ*_H_ 6.84, 6.42, 6.38 and 5.93), five methylene protons (*δ*_H_ 3.15, 2.47, *δ*_H_ 2.94, 2.22, *δ*_H_ 2.59, 2.25, *δ*_H_ 1.84, 1.63 and *δ*_H_ 1.63, 1.50), three methine protons (*δ*_H_ 3.33, 3.19 and 2.19) and five methyl groups (*δ*_H_ 1.83, 1.81, 1.79, 1.47 and 0.94) (Table 1). The combination of ^13^C NMR and HSQC spectra revealed the presence of 25 carbon resonances, including one ketone (*δ*_C_ 216.6), one aldehyde carbon (*δ*_C_ 194.0), four olefinic carbons, five methylene, three methine, five methyl and six quaternary carbons (*δ*_C_ 146.8, 142.1, 134.9, 124.9, 76.6 and 43.6) (Table 2). Spin systems and their partial structures were confirmed and assembled by combined analysis of COSY and HMBC correlations (Figure 2). Three spin systems, H_2_-1/H-2/H-6, H-8/H_2_-9/H-10 and H_2_-12/H_2_-13, and HMBC correlations from H_3_-22 (*δ*_H_ 0.94) to C-1 (*δ*_C_ 41.4), C-10 (*δ*_C_ 47.8), C-11 (*δ*_C_ 43.6) and C-12 (*δ*_C_ 44.5), and from H-21 (*δ*_H_ 9.23) to C-6 (*δ*_C_ 48.8), C-7 (*δ*_C_ 142.1) and C-8 (*δ*_C_ 158.4) confirmed the presence of an eight-membered ring system with an aldehyde group. The five-membered ring with a ketone was also determined by the HMBC correlations from H_3_-20 (*δ*_H_ 1.47) to C-2 (*δ*_C_ 49.6), C-3 (*δ*_C_ 76.6) and C-4 (*δ*_C_ 55.0) and from H-6 (*δ*_H_ 3.33) to C-4 (*δ*_C_ 55.0), C-5 (*δ*_C_ 216.6), C-7 (*δ*_C_ 142.1) and C-21 (*δ*_C_ 194.0). Additionally, the HMBC correlations from H_2_-13 (*δ*_H_ 2.25, 2.59) to C-10 (*δ*_C_ 47.8), C-14 (*δ*_C_ 146.8) and C-15 (*δ*_C_ 124.9) suggested that one additional five-membered ring was connected to the eight-membered ring, which generated a 5-8-5 tricyclic carbon skeleton. The partial structure was closely related to ophiobolin analogs and the ^1^H and ^13^C NMR spectra of **1** resembled those of 6-*epi*-ophiobolin C (**6**) except for the presence of two olefinic protons (*δ*_H_ 6.38 and 6.42) and two sp^2^ quaternary carbons (*δ*_C_ 146.8 and 124.9). Finally, the COSY correlation of H-16/H-17/H-18 and the HMBC correlations from H_3_-24 (*δ*_H_ 1.81) and H_3_-25 (*δ*_H_ 1.79) to C-18 (*δ*_C_ 125.9) and C-19 (*δ*_C_ 134.9) and from H_3_-23 (*δ*_H_ 1.83) to C-14 (*δ*_C_ 146.8), C-15 (*δ*_C_ 124.9) and C-16 (*δ*_C_ 130.6) defined a conjugated side chain connected to C-14 of the tricyclic ring. The planar structure of **1** was elucidated to possess a fully unsaturated side chain. To the best of our knowledge, **1** is the first ophiobolin with three double bonds at the side chain and is named 14,15-dehydro-6-*epi*- ophiobolin K.

**Figure 3:**
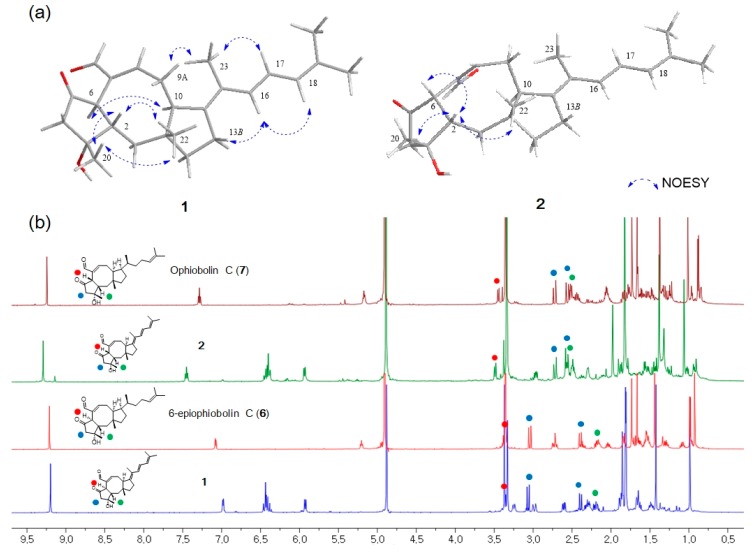
(**a**) Key NOESY correlations of **1** and **2**. (**b**) Comparison of chemical shifts of H_2_-4 and H-6 in **1** (H-6α), **2** (H-6β), **6** (H-6α) and **7** (H-6β).

The stereochemistry of **1** was determined by the analysis of proton-proton coupling constants and NOESY data. The strong NOESY correlations of H-6/H-10 and H-2/H_3_-20/H_3_-22 suggested that H-6 and H-10 were on the same face, and H-2, H_3_-20 and H_3_-22 were on opposite faces. Based on a comprehensive literature review, ophiobolin analogs have A/B-*cis* or A/B-*trans* isomers at C-2 and C-6 [18]. It has been reported that H-2 of the 6-epimer having H-6α is shielded by ca. 0.2-0.3 ppm in comparison with the A/B-cis isomer having H-6β [13]. On the basis of this analysis, the H-2 protons of **1** and **2** were observed at *δ*_H_ 2.19 and *δ*_H_ 2.57, respectively, indicating that **1** has an A/B-*trans* ring structure. The lack of a NOESY correlation of H-2/H-6 and a comparison of the ^1^H NMR data of **1** with those of 6-*epi*-ophiobolin C (**6**) also supported the fact that the A/B ring junction is *trans* in **1** (Figure 3). The relative configuration of the side chain was confirmed by comprehensive NOESY and ^1^H NMR analyses. The NOESY correlations of H-9α/H_3_-23 and H-13β/H-16 indicated the relative configuration of Δ^14,15^ was *E* conformation (Figure 3a). The geometry of the Δ^16,17^ was confirmed as *E* by the large coupling constants of H-16 (d, *J* = 15.3 Hz) and H-17 (dd, *J* = 15.3, 9.7 Hz) and NOESY correlations of H-13β/H-16, H-17/H_3_-23 and H-16/H-18. Moreover, a combination of literature review and comparison of the NMR spectral data and spectral properties of **1** with those of **6**, suggested that 14, 15-dehydro-6-*epi*-ophiobolin K (**1**) has the same absolute configuration of a 5-8-5 core structure in 6-*epi*-ophiobolin K [13,19,20].

Compound **2** had the same molecular formula C_25_H_32_O_3_ as **1**. Its ^1^H and ^13^C NMR data were similar to those of **1**, differing only by slightly shifted proton and carbon signals. It has been reported that H-2 of the 6-*epi* isomer having H-6α (A/B-*trans*) is upfield-shifted in comparison with the A/B-*cis* ophiobolin [13] (Figure 3b). The H-2 proton (*δ*_H_ 2.57) of **2** is downfield-shifted than that (*δ*_H_ 2.19) of **1**, indicating that **2** has an A/B-*cis* ring structure. This study also revealed that the chemical shifts of the geminal proton H_2_-4 are closer to each other when the A/B ring junction is *cis* than when it is *trans* (Figure 3b). The key NOE correlations of H-2/H-6, H-2/H_3_-20 and H-2/H_3_-22 suggested that **2** has an A/B-*cis* ring structure and is a stereoisomer of **1** (Figure 3a). Based on these results, the structure of **2** was determined and named 14,15-dehydro-ophiobolin K.

**Figure 4:**
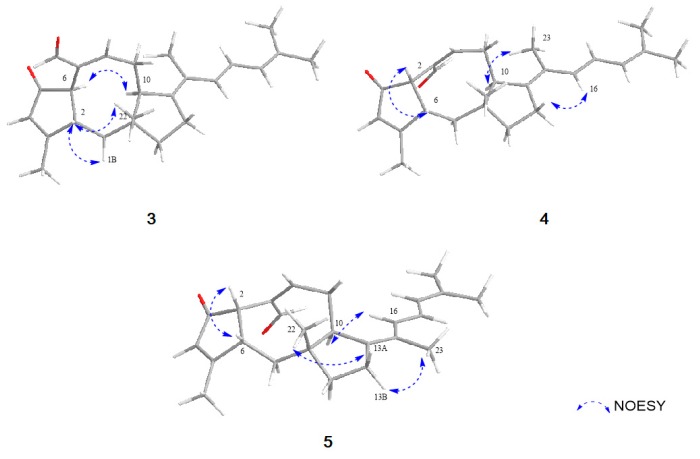
Key NOESY correlations of **3**–**5**.

Compound **3** was obtained as an amorphous powder with the molecular formula of C_25_H_32_O_2_ based on HRESIMS. The molecular formula of **3** has one less CH_2_ and one less oxygen compared to that of **1**. The ^1^H and ^13^C NMR data of **3** were quite similar to those of **1**, displaying one additional singlet olefin signal (*δ*_H_ 6.01) and a sp^2^ quaternary carbon at C-3 (*δ*_C_ 179.9), while lacking a methylene and sp3 quaternary carbon signal. The HMBC correlations from H_3_-20 (*δ*_H_ 2.12) to C-2 (*δ*_C_ 49.5), C-3 (*δ*_C_ 179.9) and C-4 (*δ*_C_ 129.4) revealed that a double bond existed between C-3 and C-4 by the dehydroxylation of the tertiary alcohol at C-3 in **1** (Figure 2). NOESY correlations from H-6/H-10 and H-2/H_3_-20/H_3_-22, the lack of NOESY correlation of H-2/H-6 and, comparison of the NMR spectral data and spectral properties of **3** with those of 6-*epi*-ophiobolin N (**8**), suggested that **3** has the same ring system as the A/B-*trans* ophiobolin [13,19] (Figure 4). On the basis of detailed data analysis, the structure of **3** was elucidated and named 14,15-dehydro-6-*epi*-ophiobolin G.

Compound **4** was isolated as an amorphous powder with the molecular formula of C_25_H_32_O_2_ as determined by HRESIMS. Its ^1^H NMR data were similar to those of **3**, differing only by slightly shifted signals. In contrast to the data for **3**, the NOESY correlation of H-2/H-6 indicated that **4** has an A/B-*cis* ring structure (Figure 4). Thus, compound **4** was identified as a stereoisomer of **3** and named 14,15-dehydro-ophiobolin G.

Compound **5** was isolated as an amorphous powder with the same molecular formula C_25_H_32_O_2_ as compound **4**, as determined by HRESIMS. The ^1^H NMR data of **5** and **4** were nearly identical except for the H-16 proton which was slightly downfield-shifted than that of **4**. By comprehensive analysis of its 1D and 2D NMR data, the planar structure of **5** was elucidated to be the same as that of **4**, differing only in the orientation of H_3_-23. The NOESY correlations of H-16/H-10 and H-23/H_2_-13 suggested the relative configuration of Δ^14,15^ in **5** was *Z* conformation, which is different from that of compound **4** (Figure 4). Therefore, the structure of **5** was elucidated to be as shown in Figure 1, and named 14, 15-dehydro-(*Z*)-14-ophiobolin G.

The structures of the four known compounds were determined as 6-*epi*-ophiobolin C (**6**), ophiobolin C (**7**), 6-*epi*-ophiobolin N (**8**) and ophiobolin N (**9**) by comparing of their ^1^H, ^13^C NMR and MS data with those reported in literature (Supplementary Materials).

The cytotoxicity of all the isolated compounds (**1**–**9**) against cancer cell lines, such as HCT-15, NUGC-3, NCI-H23, ACHN, PC-3 and MDA-MB-231, was investigated using the sulforhodamine B (SRB) assay, with adriamycin as a positive control. The results showed that all compounds were strongly active against 6 cancer cell lines with GI_50_ values in the range of 0.14 to 2.01 μM (Table 3). Compound **1** displayed the strongest cytotoxicity against the HCT-15, NUGC-3 and MDA-MB-231 cell lines with GI_50_ values of 0.21, 0.19 and 0.14 μM, respectively. Based on the cytotoxicity results, the analogs with one double bond (**6**–**9**) in the side chain seemed to be slightly more active than those with three double bonds (**1**–**5**). **5** was least active against all cell lines, even with GI_50_ values ranging from 1.53 to2.01 μM, indicating that the geometry of C-14/C-15 might appear to have a slight influence on their activities. In addition, results for all the strongly active compounds indicated that the stereochemistry of C-6 and the hydroxyl group at C-3 might not noticeably affect the cytotoxicity.

## 3. Materials and Methods

### 3.1. General Experimental Procedures

The 1D (^1^H and ^13^C) and 2D (COSY, HSQC, HMBC, and NOESY) NMR spectra were obtained on a Bruker 600 MHz spectrometer. Specific optical rotations were obtained on a Rudolph Research Analytical (Autopol III) polarimeter. UV-visible spectra were acquired on a Shimadzu UV-1650PC spectrophotometer in 1 mm quartz cells. IR spectra were recorded on a JASCO FT/IR-4100 spectrophotometer. High-resolution ESIMS were recorded on a hybrid ion-trap time-of-flight mass spectrometer (Shimadzu LC/MS-IT-TOF). HPLC was performed using a PrimeLine Binary pump with RI-101(Shodex). RP-HPLC was performed using a semi-prep ODS column (YMC-Triart C18, 250 × 10 mm i.d, 5 µm) and an analytical ODS column (YMC-Triart C18, 250 × 4.6 mm i.d, 5 µm).

### 3.2. Fungal Material and Fermentation

The fungus 168ST-16.1 was isolated from the algae *Padina* sp., collected at a depth of 10 m in Son Tra peninsular, Da Nang, Vietnam (16°09′97.8″ N, 108°29′96.1″ E), in August 2016. The fungal strain was identified as *Aspergillus flocculosus* (GenBank accession number MG920345) by DNA amplification and ITS region sequencing and named *Aspergillus flocculosus* 168ST-16.1.

The isolated fungi were cultured on rice media at 28 °C for three weeks in 100 Erlenmeyer flasks (500 mL), each containing rice (20.0 g), yeast extract (20.0 mg), KH_2_PO_4_ (10 mg), and natural sea water (40 mL).

### 3.3. Isolation of Compounds ***1***–***9***

The whole fermentation media were extracted with EtOAc and evaporated *in vacuo* to give the crude extract (22 g), which was fractionated by flash column chromatography on ODS using a gradient of MeOH/ H_2_O (1:4, 2:3, 3:2, 4:1 and 100% MeOH, each fraction 300× 3). The second fraction eluted with 100% MeOH was separated into ten subfractions (Fr. A-J) by column chromatography on ODS eluting with a step gradient of MeCN/H_2_O (70:30 to 100:0, v/v). Fr. E (200 mg) was further purified by an analytical reversed-phase HPLC (YMC-Pack-ODS-A, 250 × 4.6 mm i.d, 5 µm, flow rate 2.5 mL/ min, isocratic elution with 55% MeCN in H_2_O, RI detector) to yield **1** (7.5 mg, *t*_R_ = 18 min) and **2** (1.5 mg, *t*_R_ = 21 min). Compounds **3** (2.2 mg, *t*_R_ = 30 min), **4** (3.1 mg, *t*_R_ = 33 min), **5** (1.2 mg, *t*_R_ = 36 min), **6** (3.5 mg, *t*_R_ = 50 min) and **7** (3.2 mg, *t*_R_ = 53 min) were isolated from Fr. F (210 mg) by a semi-preparative reversed-phase HPLC (YMC-Pack-ODS-A, 250 × 10 mm i.d, 5 µm, flow rate 6.0 mL/ min, isocratic elution with 60% MeCN in H_2_O, RI detector). Fr G (136 mg) was subjected to a semi-preparative reversed-phase HPLC (YMC-Pack-ODS-A, 250 × 10 mm i.d, 5 µm, flow rate 5.5 mL/ min, isocratic elution with 65% MeCN in H_2_O, RI detector) to obtain **8** (3.6 mg, *t*_R_ = 40 min) and **9** (2.9 mg, *t*_R_ = 43 min).

14,15-dehydro-6-*epi*-ophiobolin K (**1**): amorphous powder; ${\left[\alpha \right]}_{D}^{23}$ +74.0(c 1.0, MeOH); IR ν_max_ 3442, 2931, 2852, 1736, 1683, 1640, 1454, 1379 cm^−1^; UV(MeOH) λ_max_ (log ε) 286 (3.62), 236 (3.04) nm; HRESIMS *m/z* 405.2405 [M + Na]^+^ (calcd for 405.2406, C_25_H_34_O_3_Na); ^1^H NMR (CDCl_3_, 600 MHz) and ^13^C NMR (CDCl_3_, 150 MHz) see Table 1.

14,15-dehydro-ophiobolin K (**2**): amorphous powder; ${\left[\alpha \right]}_{D}^{23}$ +94.0(c 1.0, MeOH); IR ν_max_ 3451, 2967, 2897, 1734, 1688, 1448, 1377, 1233 cm^−1^; UV(MeOH) λ_max_ (log ε) 289 (3.58), 238 (3.36) nm; HRESIMS *m/z* 405.2404 [M + Na]^+^ (calcd for 405.2406, C_25_H_34_O_3_Na); ^1^H NMR (CD_3_OD, 600 MHz) and ^13^C NMR (CD_3_OD, 150 MHz) see Table 1.

14,15-dehydro-6-*epi*-ophiobolin G (**3**): amorphous powder; ${\left[\alpha \right]}_{D}^{23}$ +87.0(c 1.0, MeOH); IR ν_max_ 2922, 2858, 1683, 1625, 1455, 1377 cm^−1^; UV(MeOH) λ_max_ (log ε) 286 (3.53), 227 (3.24) nm; HRESIMS *m/z* 387.2301 [M + Na]^+^ (calcd for 387.2300, C_25_H_32_O_2_Na); ^1^H NMR (CD_3_OD, 600 MHz) and ^13^C NMR (CD_3_OD, 150 MHz) see Table 1.

14,15-dehydro-ophiobolin G (**4**): amorphous powder; ${\left[\alpha \right]}_{D}^{23}$ +85.0(c 1.0, MeOH); IR ν_max_ 2925, 2858, 1689, 1636, 1441, 1377 cm^−1^; UV(MeOH) λ_max_ (log ε) 291 (3.59), 231 (3.37) nm; HRESIMS *m/z* 387.2299 [M + Na]^+^ (calcd for 387.2300, C_25_H_32_O_2_Na); ^1^H NMR (CD_3_OD, 600 MHz) and ^13^C NMR (CD_3_OD, 150 MHz) see Table 1.

14,15-dehydro-(*Z*)-14-ophiobolin G (**5**): amorphous powder; ${\left[\alpha \right]}_{D}^{23}$ +132.0(c 1.0, MeOH); IR ν_max_ 2922, 2855, 1692, 1632, 1437, 1377 cm^−1^; UV(MeOH) λ_max_ (log ε) 289 (3.61), 231 (3.54) nm; HRESIMS *m/z* 387.2299 [M + Na]^+^ (calcd for 387.2300, C_25_H_32_O_2_Na); ^1^H NMR (CD_3_OD, 600 MHz) and ^13^C NMR (CD_3_OD, 150 MHz) see Table 1.

### 3.4. Cytotoxicity Test by SRB Assay

The human cancer cell lines, HCT-15 (colon), NUGC-3 (stomach), NCI-H23 (lung), ACHN (renal), PC-3 (prostate) and MDA-MB-231 (breast), were purchased from American Type Culture Collection (Manassas, VA, USA). They were then cultured in RPMI 1640 supplemented with 10% fetal bovine serum (FBS). Cell cultures were maintained at 37 °C under a humidified atmosphere of 5% CO_2_. The growth inhibition assay against human cancer cell lines was performed in accordance with the sulforhodamine B (SRB) assay [22]. In brief, 8,000 cells/well were seeded onto a 96-well plate. On the following, the cells were treated with compounds **1**–**9**, vehicle control (0.1% DMSO) and positive control (adriamycin). After incubation for 48 h, the cultures were fixed with 50% trichloroactetic acid (50 μg/mL) and stained with 0.4% sulforhodamine B in 1% acetic acid. Unbound dye was removed by washing with 1% acetic acid, and protein-bound dye was extracted with 10 mM Tris base (pH 10.5) for optical density determination. Absorbance at 540 nm was determined using a VersaMax microplate reader from Molecular Devices (LLC, Sunnyvale, CA, USA). GI_50_ values were calculated using GraphPad Prism 4.0 software from GraphPad Software, Inc. (San Diego, CA, USA).

## 4. Conclusions

Chemical investigation of the marine-derived fungus *Aspergillus flocculosus* 168ST-16.1 led to the isolation and identification of five new (**1**–**5**) and four known (**6**–**9**) ophiobolin derivatives. The five new ophiobolins possessed a fully unsaturated side chain, and **5** had a Z-conformation at C-14/C-15. To the best of our knowledge, the new compounds **1**–**5** are the first ophiobolins with three double bonds at the side chain. All compounds (**1**–**9**) exhibited potent growth inhibitory activities against the HCT-15, NUGC-3, NCI-H23, ACHN, PC-3 and MDA-MB-231 cancer cell lines. The cytotoxicities of the new ophiobolins **4** and **5** were slightly weaker or similar to those of the known compounds (**6**–**9**). These results suggest that dehydration at C-14 and C-15 might not significantly affect the cytotoxicity against cancer cell lines. This study is the first report to describe the effect of the side chain of ophiobolins by evaluating the anticancer activity of five new and four known compounds together.

## Figures and Tables

**Table 1:** ^1^H and ^13^C NMR data for **1**, **2** and **3** at 600 MHz (*δ* in ppm, *J* in Hz).

Position	1 ^a^	2 ^b^	3 ^b^	4 ^b^	5 ^b^
1α	1.63, m	1.54, m	1.22 (t, 13.2)	1.44, m	1.42, m
1β	1.84, m	1.54, m	2.15 (dd, 13.2, 3.6)	2.13 (dd, 15.8, 4.5)	2.13 (dd, 15.8, 4.5)
2	2.19, m	2.57, m	2.72 (d, 12.9)	3.32, overlap	3.31, overlap
4	2.47 (d, 16.6)	2.57 (d, 18.9)	6.01, s	6.09, s	6.08, s
	3.15 (d, 16.6)	2.70 (d, 18.9)			
6	3.33 (d, 10.5)	3.47 (d, 11.8)	3.53 (d, 3.6)	4.29 (d, 7.2)	4.28 (d, 7.2)
8	6.84 (d, 6.7)	7.44 (t, 8.6)	6.91 (dd, 6.2, 2.3)	7.24 (d, 6.7)	7.21 (d, 6.8)
9α	2.94 (d, 20.8)	2.48, m	3.02 (d, 21.3)	2.37, m	2.30, m
9β	2.22, m	2.48, m	2.31, m	2.37, m	2.22, m
10	3.19 (d, 13.0)	2.28, m	3.37 (d, 13.1)	3.03 (d, 16.5)	3.06 (d, 16.5)
12α	1.50, m	1.43, m	1.45, m	1.46, m	1.44, m
12β	1.63, m	1.86, m	1.65, m	1.46, m	1.44, m
13α	2.25 (dd, 14.8, 6.3)	2.48, m	2.31, m	2.40, m	2.37, m
13β	2.59, m	2.95, m	2.58 (dd, 14.8, 6.4)	3.08, m	3.03, m
16	6.42 (d, 15.3)	6.37 (d, 15.1)	6.43 (d, 15.3)	6.31 (d, 15.3)	6.14 (d, 15.1)
17	6.38 (dd, 15.3, 9.7)	6.39 (dd, 15.1, 10.1)	6.40(dd, 15.3, 9.3)	6.34 (dd, 15.3, 10.3)	6.31 (dd, 15.1, 10.5)
18	5.93 (d, 9.7)	5.92 (d, 10.1)	5.90 (d, 9.3)	5.88 (d, 10.3)	5.94 (d, 10.5)
20	1.47, s	1.37, s	2.12, s	2.29, s	2.27, s
21	9.23, s	9.28, s	9.25, s	9.46, s	9.44, s
22	0.94, s	1.05, s	0.99, s	0.86, s	0.84, s
23	1.83, s	1.96, s	1.83, s	1.72, s	1.72, s
24	1.81, s	1.81, s	1.78, s	1.79, s	1.77, s
25	1.79, s	1.81, s	1.79, s	1.80, s	1.80, s

The assignments were aided by COSY, NOESY, HSQC, and HMBC NMR spectra. ^a^ Measured in CDCl_3_; ^b^ Measured in methanol-*d*_4_.

**Table 2:** ^13^C NMR data for **1**–**5** at 150 MHz (*δ* in ppm).

Position	1 ^a^	2 ^b^	3 ^b^	4 ^b^	5 ^b^
1	41.4	41.5	45.3	34.9	34.9
2	49.6	50.6	49.5	49.8	49.9
3	76.7	76.6	179.9	180.2	180.2
4	55.0	53.9	129.4	130.1	130.1
5	216.6	216.2	209.4	209.2	209.4
6	48.8	48.8	49.7	48.0	48.0
7	142.1	141.8	140.6	138.7	138.6
8	158.4	160.7	156.7	159.7	160.0
9	34.1	29.3	34.0	28.8	29.9
10	47.8	56.2	47.2	41.9	40.7
11	43.6	44.2	44.0	45.1	45.3
12	44.5	34.9	43.4	40.4	40.4
13	27.2	26.9	26.8	31.4	33.1
14	146.8	143.3	146.6	143.2	142.7
15	124.9	126.5	125.0	127.1	126.6
16	130.6	131.1	130.6	130.0	129.6
17	123.6	122.7	123.2	122.9	123.9
18	125.9	126.0	126.0	126.0	126.1
19	134.9	133.7	133.5	133.6	133.8
20	25.8	24.4	15.7	17.2	17.1
21	194.0	195.1	193.2	195.1	195.2
22	21.3	18.0	19.8	24.6	24.7
23	13.6	14.1	13.8	14.2	15.5
24	18.4	16.9	16.9	16.9	16.9
25	26.1	24.8	24.8	24.8	24.8

^a^ Measured in CDCl_3_; ^b^ Measured in methanol-*d*_4_.

**Table 3:** Growth Inhibition (GI_50_, μM) Values of **1**–**9** against Human Tumor Cell Lines.

Cell Lines ^a^	GI_50_ (μM)									
	1	2	3	4	5	6	7	8	9	ADR ^b^
HCT-15	0.21	0.44	0.96	1.24	1.67	0.24	0.21	0.30	0.22	0.13
NUGC-3	0.19	0.50	0.88	1.07	1.53	0.22	0.20	0.22	0.20	0.15
NCI-H23	0.18	0.61	1.40	1.50	1.84	0.24	0.16	0.22	0.22	0.15
ACHN	0.24	0.53	1.14	1.40	2.01	0.43	0.20	0.23	0.42	0.16
PC-3	0.24	0.47	1.00	1.38	1.60	0.27	0.36	0.20	0.20	0.14
MDA-MB-231	0.14	0.63	1.05	1.35	1.75	0.19	0.22	0.21	0.19	0.15

^a^ HCT-15: Colon cancer, NUGC-3: Stomach cancer, NCI-H23: Lung cancer, ACHN: Renal cancer, PC-3: Prostate cancer, MDA-MB-231: Breast cancer; GI_50_ values are the concentration corresponding to 50% growth inhibition. ^b^ ADR: Adriamycin as standard.

## Supplementary Materials

The followings are available online at https://www.mdpi.com/1660-3397/17/6/346/s1, Figures S1–S35: HRESI-MS data, ^1^H NMR, ^13^C NMR, COSY, HSQC, HMBC, NOESY and experimental spectra of **1**–**5**, Figures S36–S43: LRMS data, ^1^H NMR, ^13^C NMR and experimental spectra of **6**–**9**.
