# Supplementary material for: New Ophiobolin Derivatives from the Marine Fungus Aspergillus flocculosus and Their Cytotoxicities against Cancer Cells
Source: Mar Drugs. 2019 Jun 11;17(6):346. doi: 10.3390/md17060346 (PMC6628216; doi:10.3390/md17060346)
Supplement: Supplementary file 1 [file marinedrugs-17-00346-s001.pdf]

Supplementary data

New Ophiobolin Derivatives from the Marine Fungus *Aspergillus flocculosus* and Their Cytotoxicities against Cancer Cells

Byeoung-Kyu Choi [1,2], Phan Thi Hoai Trinh [3,4], Hwa-Sun Lee [2], Byeong-Woo Choi [2], Jong Soon Kang [5], Ngo Thi Duy Ngoc [3], Tran Thi Thanh Van [3,4] and Hee Jae Shin [1,2,*]

1 Department of Marine Biotechnology, University of Science and Technology (UST), 217 Gajungro, Yuseong-gu, Daejeon 34113, Korea; choibk4404@kiost.ac
2 Marine Natural Products Chemistry Laboratory, Korea Institute of Ocean Science and Technology, 385 Haeyang-ro, Yeongdo-gu, Busan 49111, Korea; hwasunlee@kiost.ac (H.-S.L.); choibw0924@gmail.com (B.-W.C.)
3 Nhatrang Institute of Technology Research and Application, Vietnam Academy of Science and Technology, 02 Hung Vuong, Nha Trang 650000, Vietnam; phanhoaitrinh84@gmail.com (P.T.H.T.); ngoduyngoc@nitra.vast.vn (N.T.D.N); tranthanhvan@nitra.vast.vn (T.T.T.V.)
4 Graduate University of Science and Technology, Vietnam Academy of Science and Technology, 18 Hoang Quoc Viet, Cau Giay, Ha Noi 100000, Vietnam
5 Laboratory Animal Resource Center, Korea Research Institute of Bioscience and Biotechnology, 30 Yeongudanjiro, Cheongju 28116, Korea; kanjon@kribb.re.kr
* Correspondence: shinhj@kiost.ac.kr; Tel.: +82-51-664-3341; Fax: +82-51-664-3340

Contents

Figure S1. HRESIMS data of 14,15-dehydro-6-*epi*-ophiobolin K (**1**). --------------------------4
Figure S2. ^{1}H NMR spectrum of 14,15-dehydro-6-*epi*-ophiobolin K (**1**). --------------------5
Figure S3. ^{13}C NMR spectrum of 14,15-dehydro-6-*epi*-ophiobolin K (**1**). -------------------6
Figure S4. ^{1}H-^{1}H COSY spectrum of 14,15-dehydro-6-*epi*-ophiobolin K (**1**). -------------7
Figure S5. HSQC spectrum of 14,15-dehydro-6-*epi*-ophiobolin K (**1**). ------------------------8
Figure S6. HMBC spectrum of 14,15-dehydro-6-*epi*-ophiobolin K (**1**). -----------------------9
Figure S7. NOESY spectrum of 14,15-dehydro-6-*epi*-ophiobolin K (**1**). -------------------10
Figure S8. HRESIMS data of 14,15-dehydro-ophiobolin K (**2**). --------------------------------11
Figure S9. ^{1}H NMR spectrum of 14,15-dehydro-ophiobolin K (**2**). ---------------------------12
Figure S10. ^{13}C NMR spectrum of 14,15-dehydro-ophiobolin K (**2**). -------------------------13
Figure S11. ^{1}H-^{1}H COSY spectrum of 14,15-dehydro-ophiobolin K (**2**). -------------------14
Figure S12. HSQC spectrum of 14,15-dehydro-ophiobolin K (**2**). ------------------------------15
Figure S13. HMBC spectrum of 14,15-dehydro-ophiobolin K (**2**). ----------------------------16
Figure S14. NOESY spectrum of 14,15-dehydro-ophiobolin K (**2**). ---------------------------17
Figure S15. HRESIMS data of 14,15-dehydro-6-*epi*-ophiobolin G (**3**). ---------------------18
Figure S16. ^{1}H NMR spectrum of 14,15-dehydro-6-*epi*-ophiobolin G (**3**). ----------------19
Figure S17. ^{13}C NMR spectrum of 14,15-dehydro-6-*epi*-ophiobolin G (**3**). ---------------20
Figure S18. ^{1}H-^{1}H COSY spectrum of 14,15-dehydro-6-*epi*-ophiobolin G (**3**). ----------21
Figure S19. HSQC spectrum of 14,15-dehydro-6-*epi*-ophiobolin G (**3**). --------------------22
Figure S20. HMBC spectrum of 14,15-dehydro-6-*epi*-ophiobolin G (**3**). -------------------23
Figure S21. NOESY spectrum of 14,15-dehydro-6-*epi*-ophiobolin G (**3**). -----------------24
Figure S22. HRESIMS data of 14,15-dehydro-ophiobolin G (**4**). ------------------------------25
Figure S23. ^{1}H NMR spectrum of 14,15-dehydro-ophiobolin G (**4**). --------------------------26
Figure S24. ^{13}C NMR spectrum of 14,15-dehydro-ophiobolin G (**4**). ------------------------27
Figure S25. ^{1}H-^{1}H COSY spectrum of 14,15-dehydro-ophiobolin G (**4**). -------------------28
Figure S26. HSQC spectrum of 14,15-dehydro-ophiobolin G (**4**). ------------------------------29
Figure S27. HMBC spectrum of 14,15-dehydro-ophiobolin G (**4**). ----------------------------30
Figure S28. NOESY spectrum of 14,15-dehydro-ophiobolin G (**4**). ---------------------------31

Figure S29. HRESIMS data of 14,15-dehydro-(*Z*)-14-ophiobolin G (**5**). --------------------32
Figure S30. ^{1}H NMR spectrum of 14,15-dehydro-(*Z*)-14-ophiobolin G (**5**). ----------------33
Figure S31. ^{13}C NMR spectrum of 14,15-dehydro-(*Z*)-14-ophiobolin G (**5**). ---------------34
Figure S32. ^{1}H-^{1}H COSY spectrum of 14,15-dehydro-(*Z*)-14-ophiobolin G (**5**). ---------35
Figure S33. HSQC spectrum of 14,15-dehydro-(*Z*)-14-ophiobolin G (**5**). -------------------36
Figure S34. HMBC spectrum of 14,15-dehydro-(*Z*)-14-ophiobolin G (**5**). ------------------37
Figure S35. NOESY spectrum of 14,15-dehydro-(*Z*)-14-ophiobolin G (**5**). ----------------38
Figure S36. LRMS data of 6-*epi*-ophiobolin C (**6**). ---39
Figure S37. ^{1}H NMR spectrum of 6-*epi*-ophiobolin C (**6**). ---39
Figure S38. LRMS data of Ophiobolin C (**7**). ---40
Figure S39. ^{1}H NMR spectrum of Ophiobolin C (**7**). --40
Figure S40. LRMS data of 6-*epi*-ophiobolin N (**8**). ---41
Figure S41. ^{1}H NMR spectrum of 6-*epi*-ophiobolin N (**8**). ---41
Figure S42. LRMS data of Ophiobolin N (**9**). ---42
Figure S43. ^{1}H NMR spectrum of Ophiobolin N (**9**). --42

Elemental Composition Report

Single Mass Analysis
Tolerance = 5.0 PPM / DBE: min = -1.5, max = 50.0
Element prediction: Off
Number of isotope peaks used for i-FIT = 3

Monoisotopic Mass, Even Electron Ions
41 formula(e) evaluated with 1 results within limits (all results (up to 1000) for each mass)
Elements Used:
C: 1-30 H: 1-50 O: 1-5 Na: 0-1

Minimum:				-1.5				
Maximum:		500.0	5.0	50.0				
Mass	Calc. Mass	mDa	PPM	DBE	i-FIT	Norm	Conf(%)	Formula
405.2405	405.2406	-0.1	-0.2	8.5	953.0	n/a	n/a	C25 H34 O3 Na

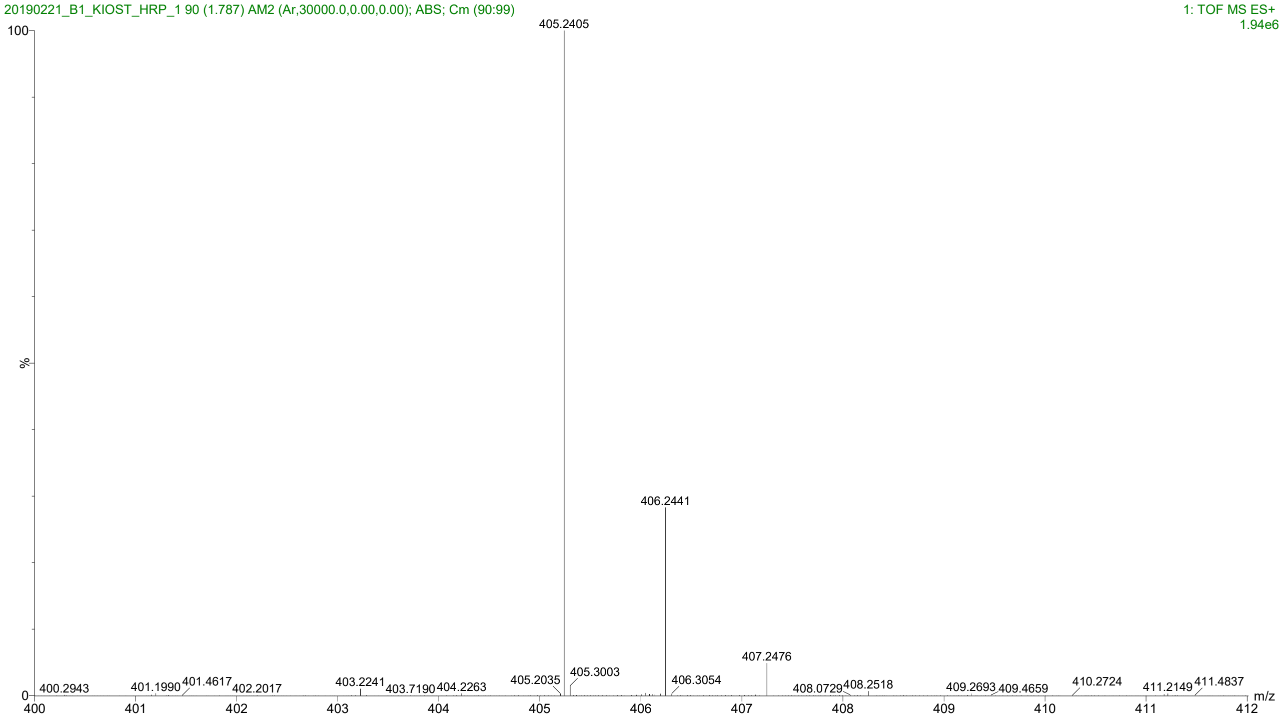

Figure S1. HRESIMS data of 14,15-dehydro-6-*epi*-ophiobolin K (**1**).

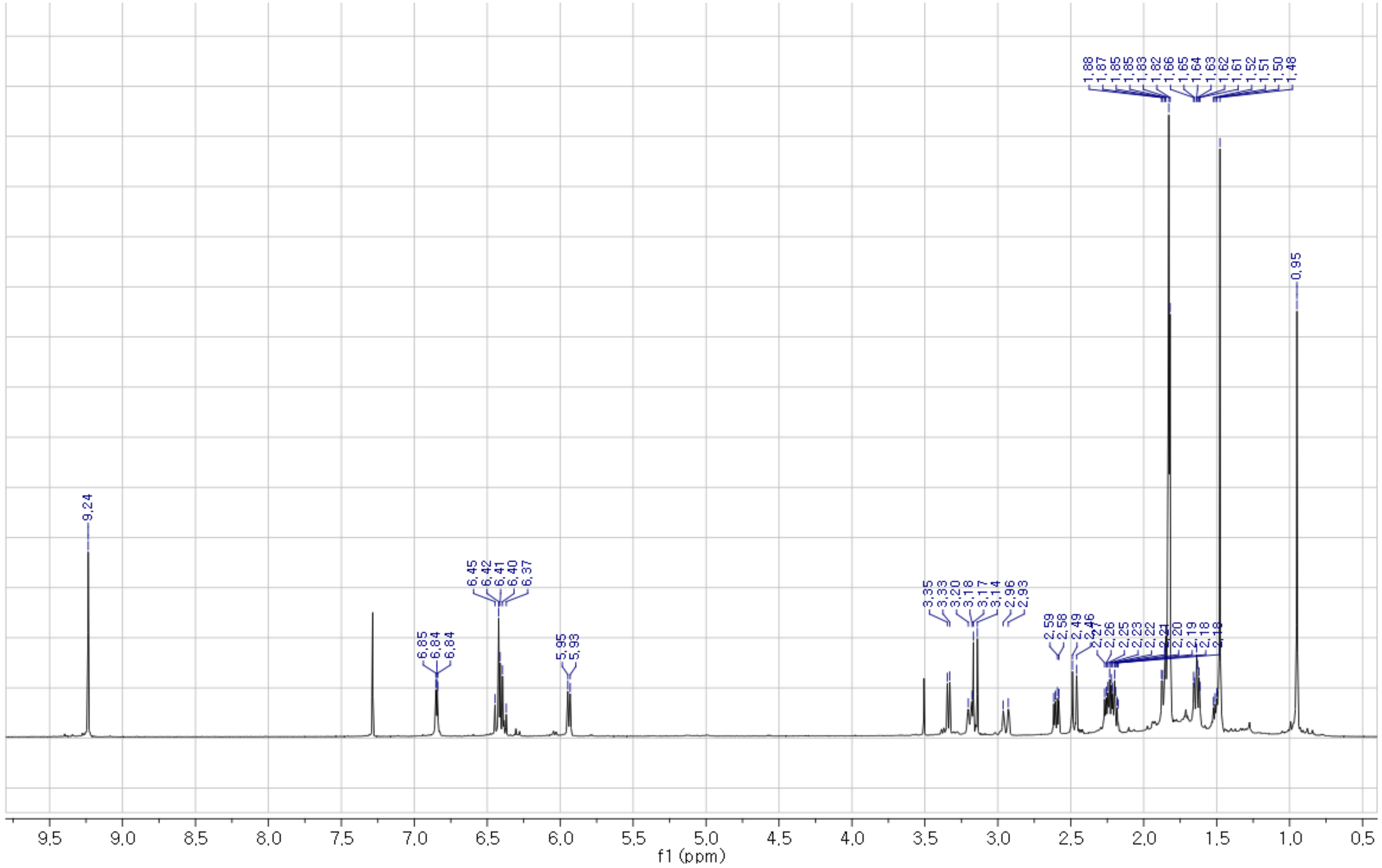

Figure S2. ^{1}H NMR spectrum of 14,15-dehydro-6-*epi*-ophiobolin K (**1**).

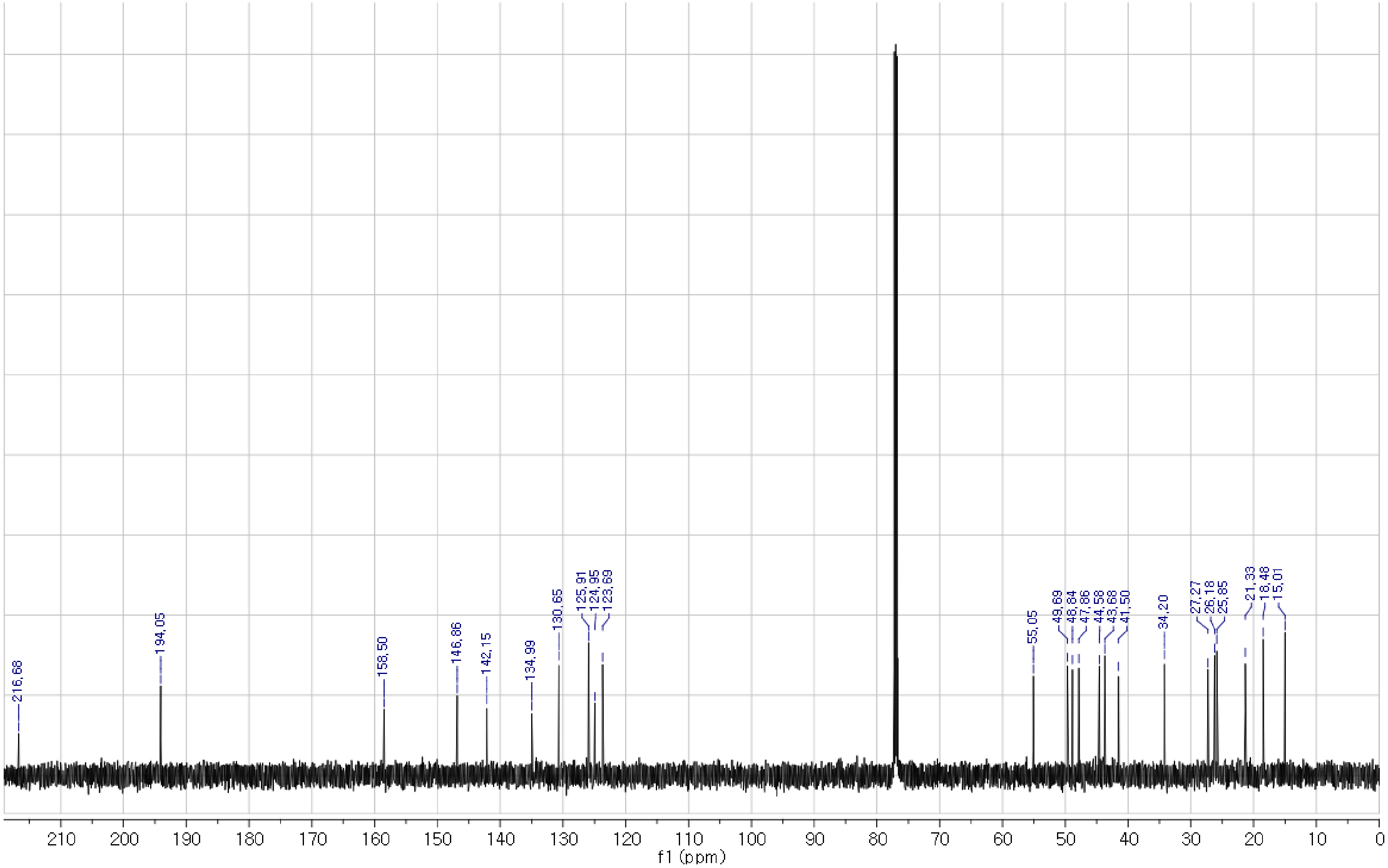

Figure S3. ^{13}C NMR spectrum of 14,15-dehydro-6-*epi*-ophiobolin K (**1**).

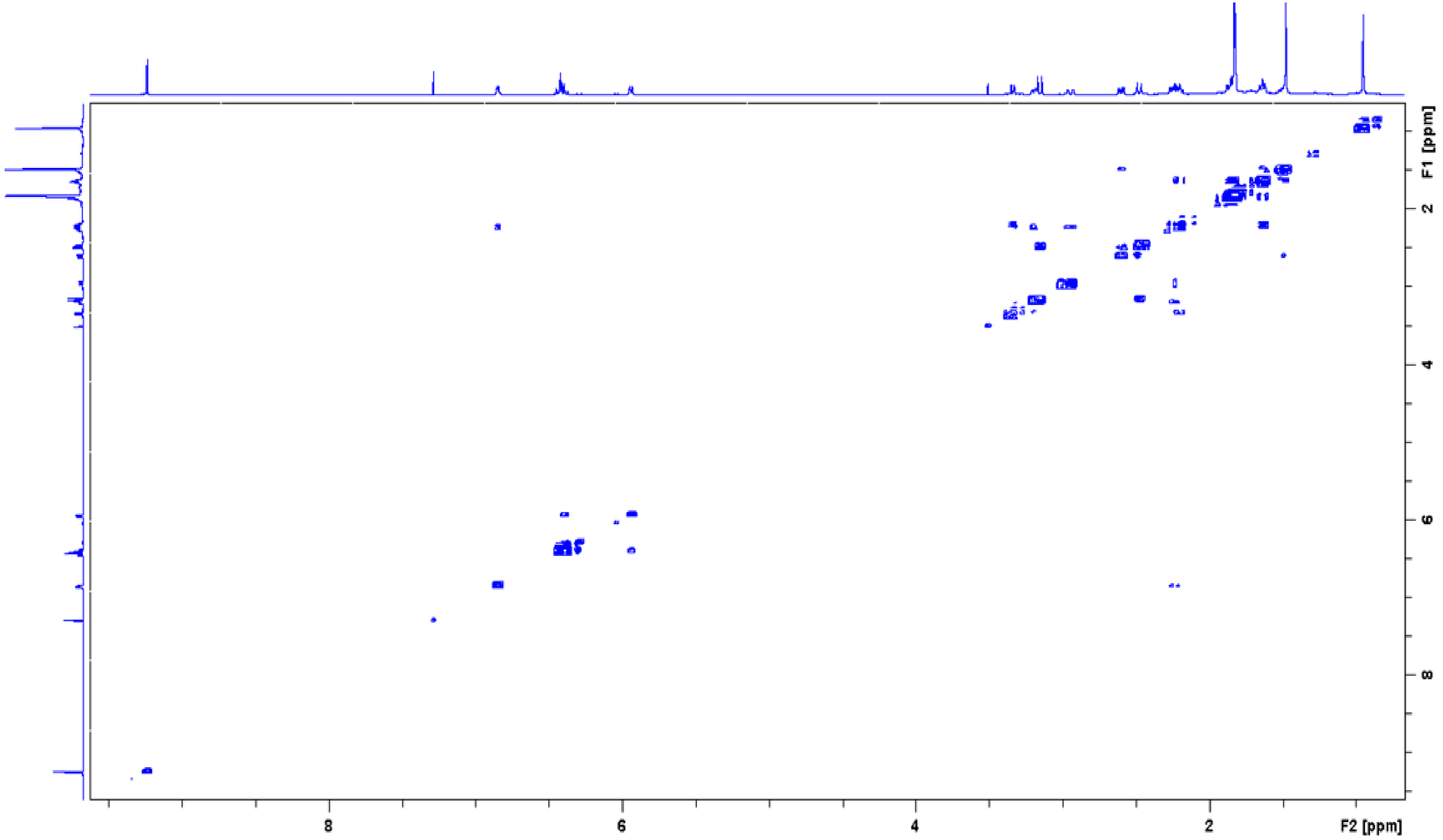

Figure S4. ^{1}H-^{1}H COSY spectrum of 14,15-dehydro-6-*epi*-ophiobolin K (**1**).

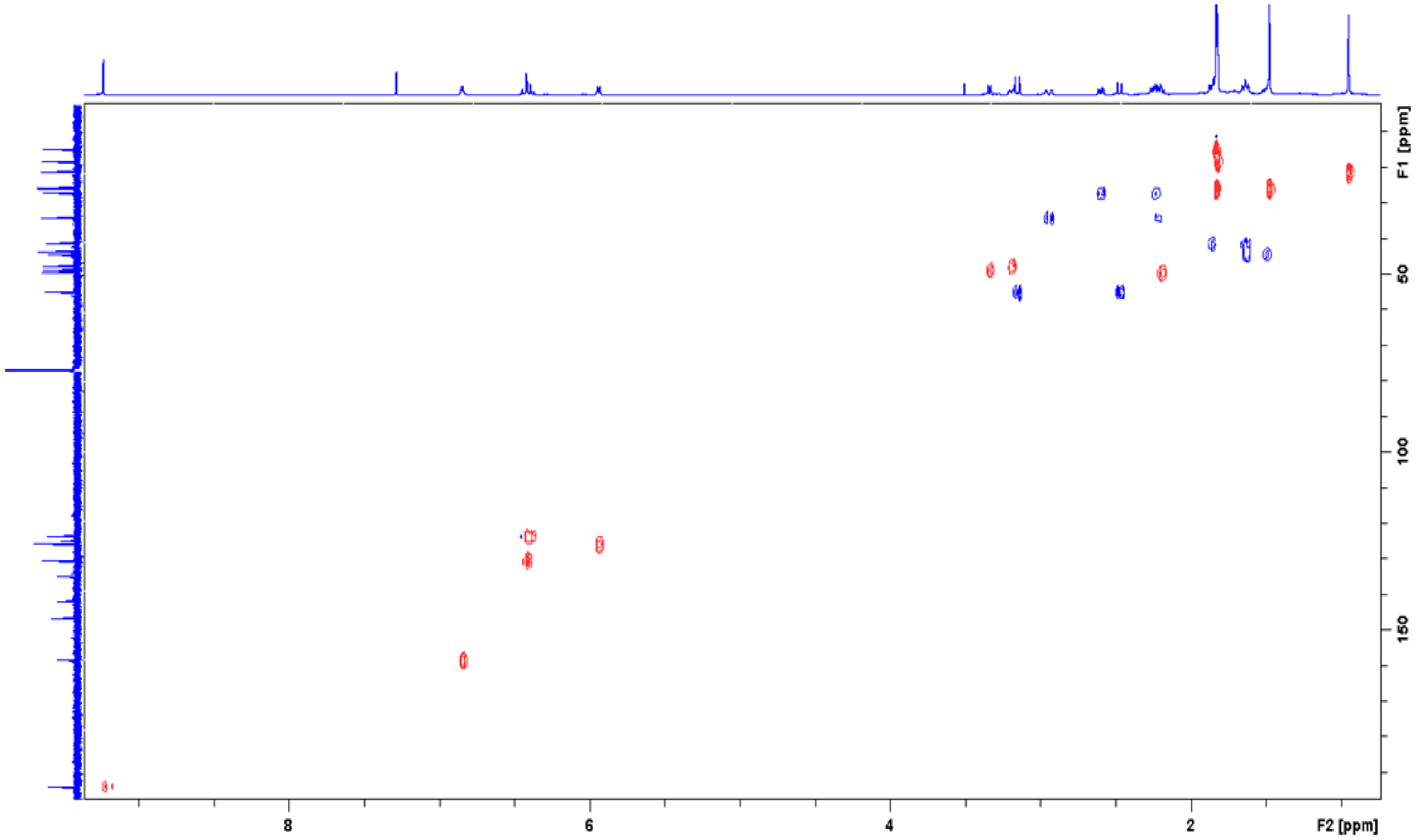

Figure S5. HSQC spectrum of 14,15-dehydro-6-*epi*-ophiobolin K (**1**).

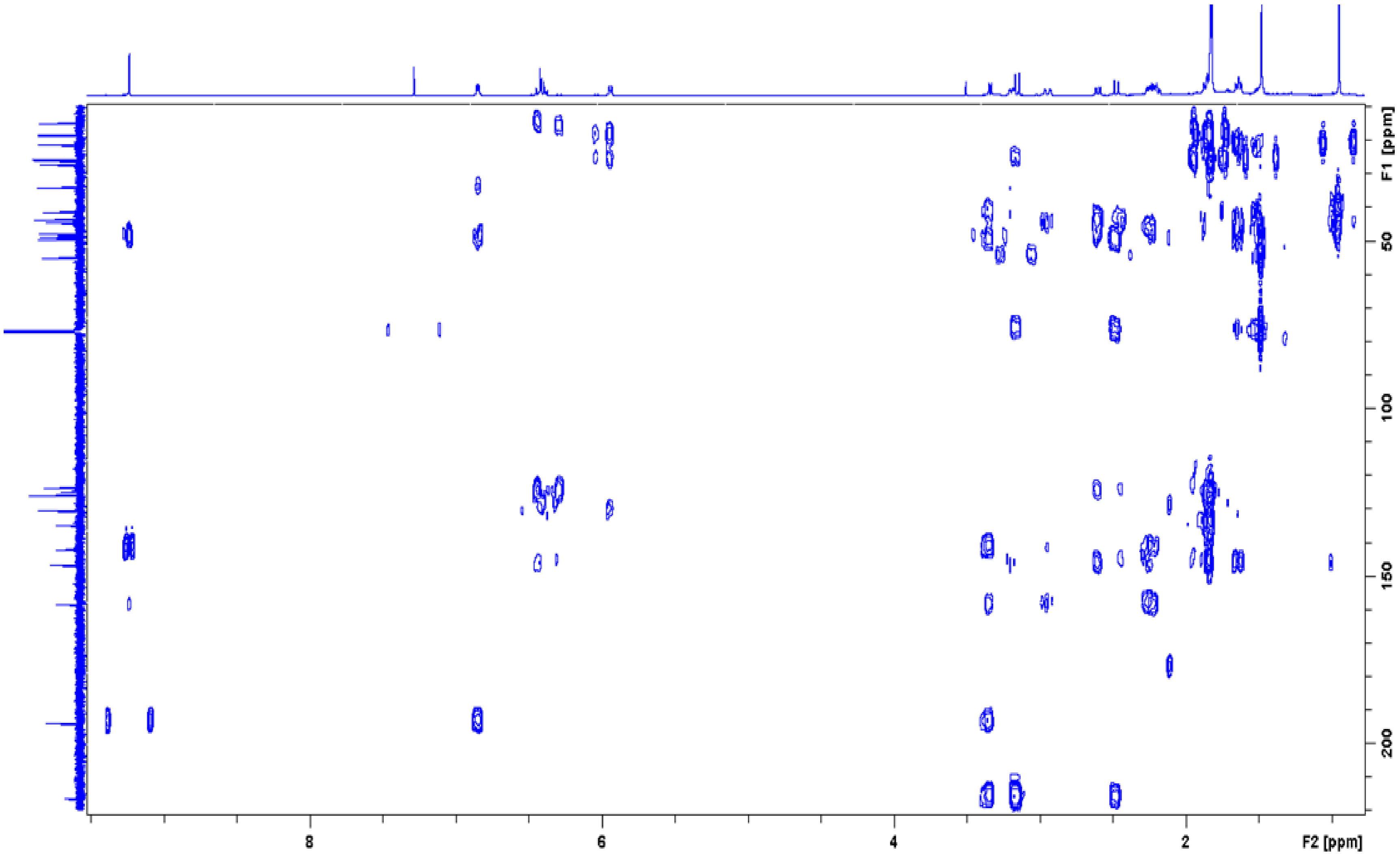

Figure S6. HMBC spectrum of 14,15-dehydro-6-*epi*-ophiobolin K (**1**).

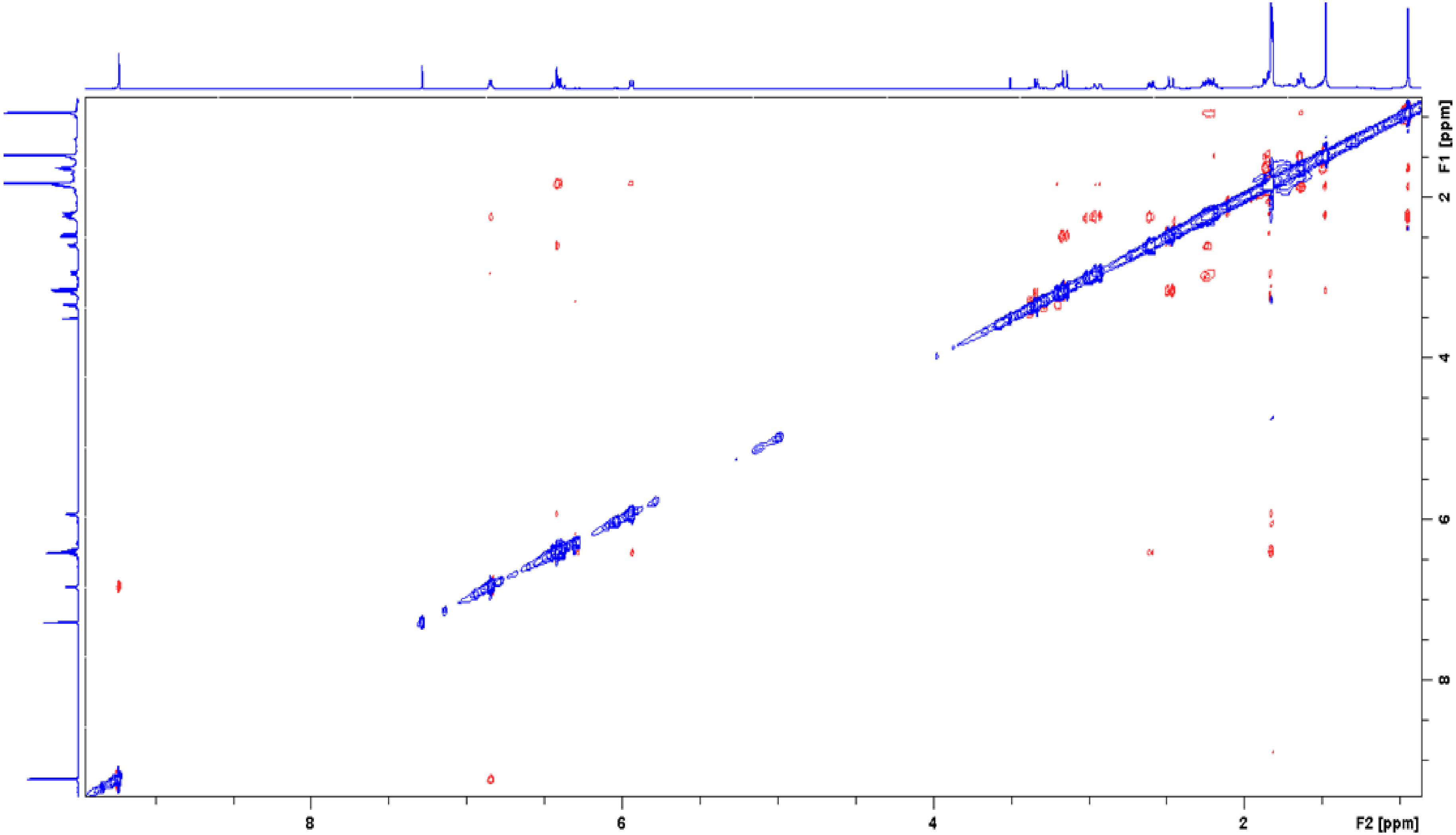

Figure S7. NOESY spectrum of 14,15-dehydro-6-*epi*-ophiobolin K (**1**).

Elemental Composition Report

Single Mass Analysis
Tolerance = 5.0 PPM / DBE: min = -1.5, max = 50.0
Element prediction: Off
Number of isotope peaks used for i-FIT = 3

Monoisotopic Mass, Even Electron Ions
41 formula(e) evaluated with 1 results within limits (all results (up to 1000) for each mass)
Elements Used:
C: 1-30 H: 1-50 O: 1-5 Na: 0-1

Minimum:				-1.5				
Maximum:		500.0	5.0	50.0				
Mass	Calc. Mass	mDa	PPM	DBE	i-FIT	Norm	Conf(%)	Formula
405.2404	405.2406	-0.2	-0.5	8.5	1146.9	n/a	n/a	C25 H34 O3 Na

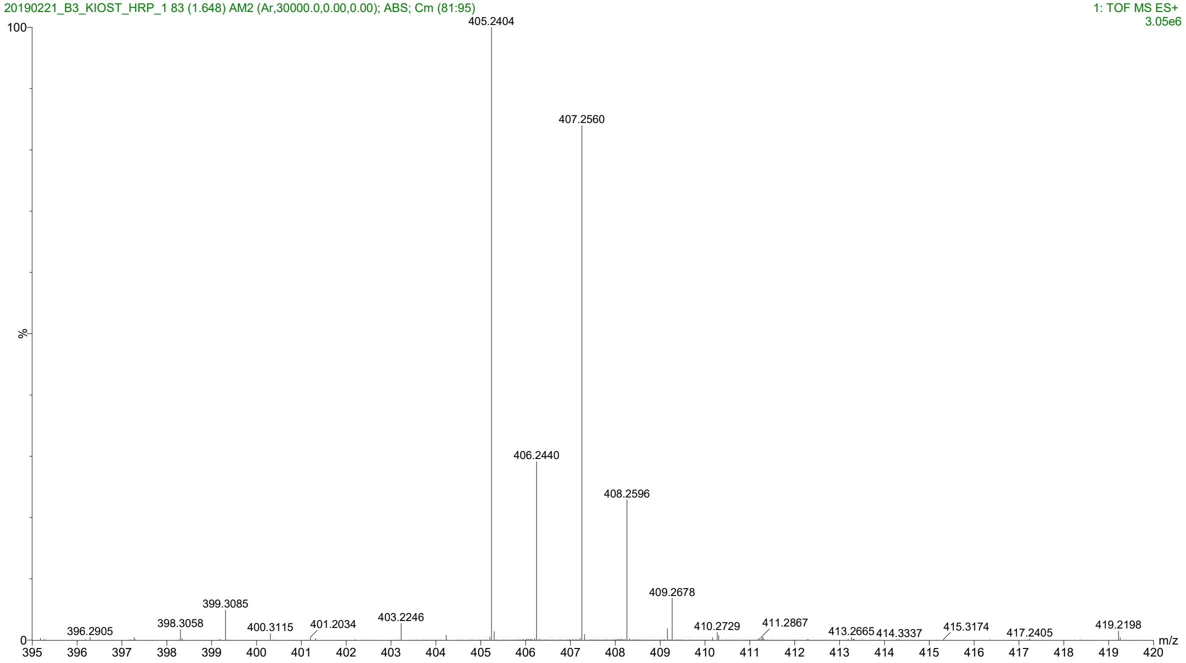

Figure S8. HRESIMS data of 14,15-dehydro-ophiobolin K (**2**).

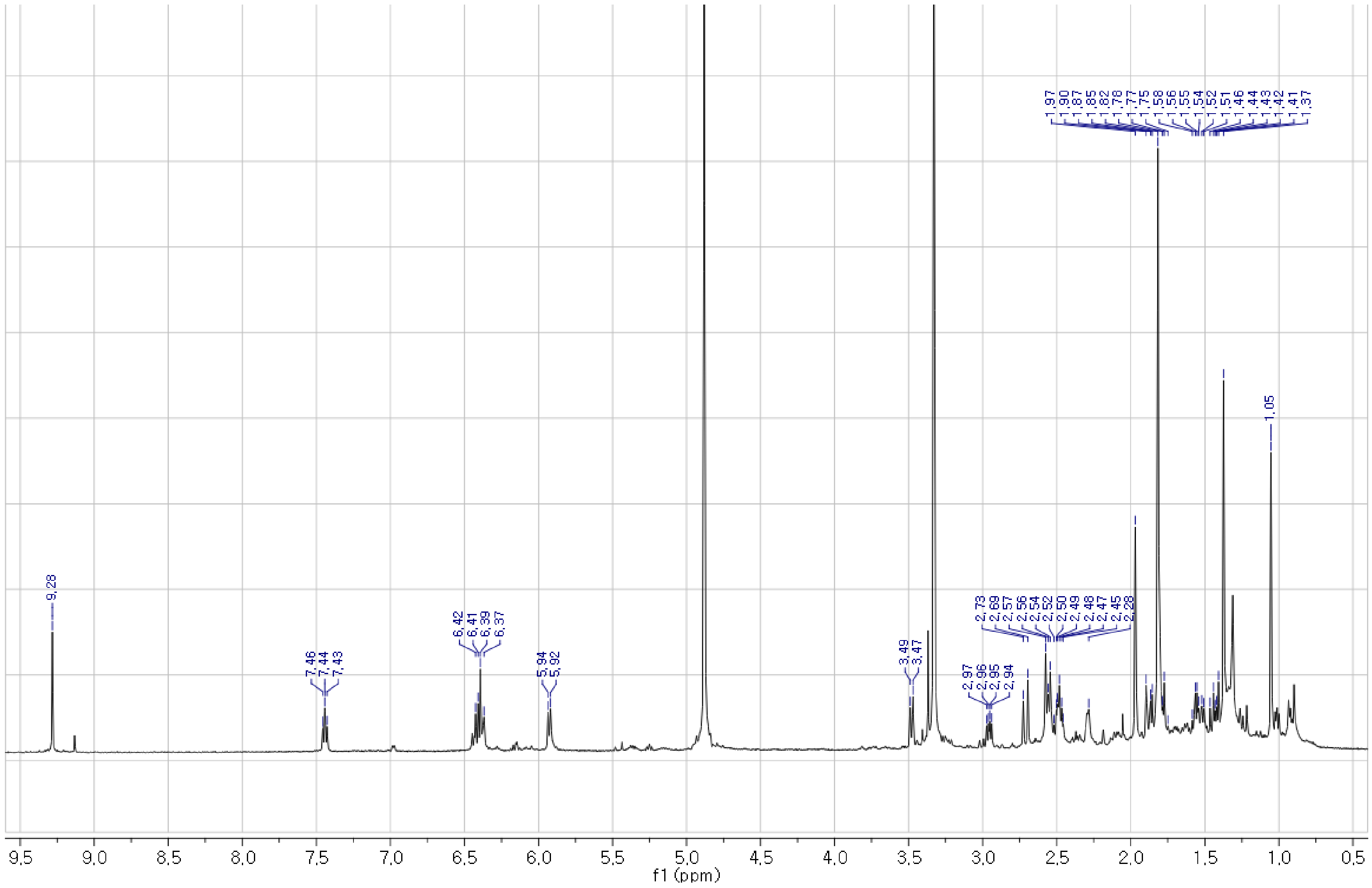

Figure S9. ^{1}H NMR spectrum of 14,15-dehydro-ophiobolin K (**2**).

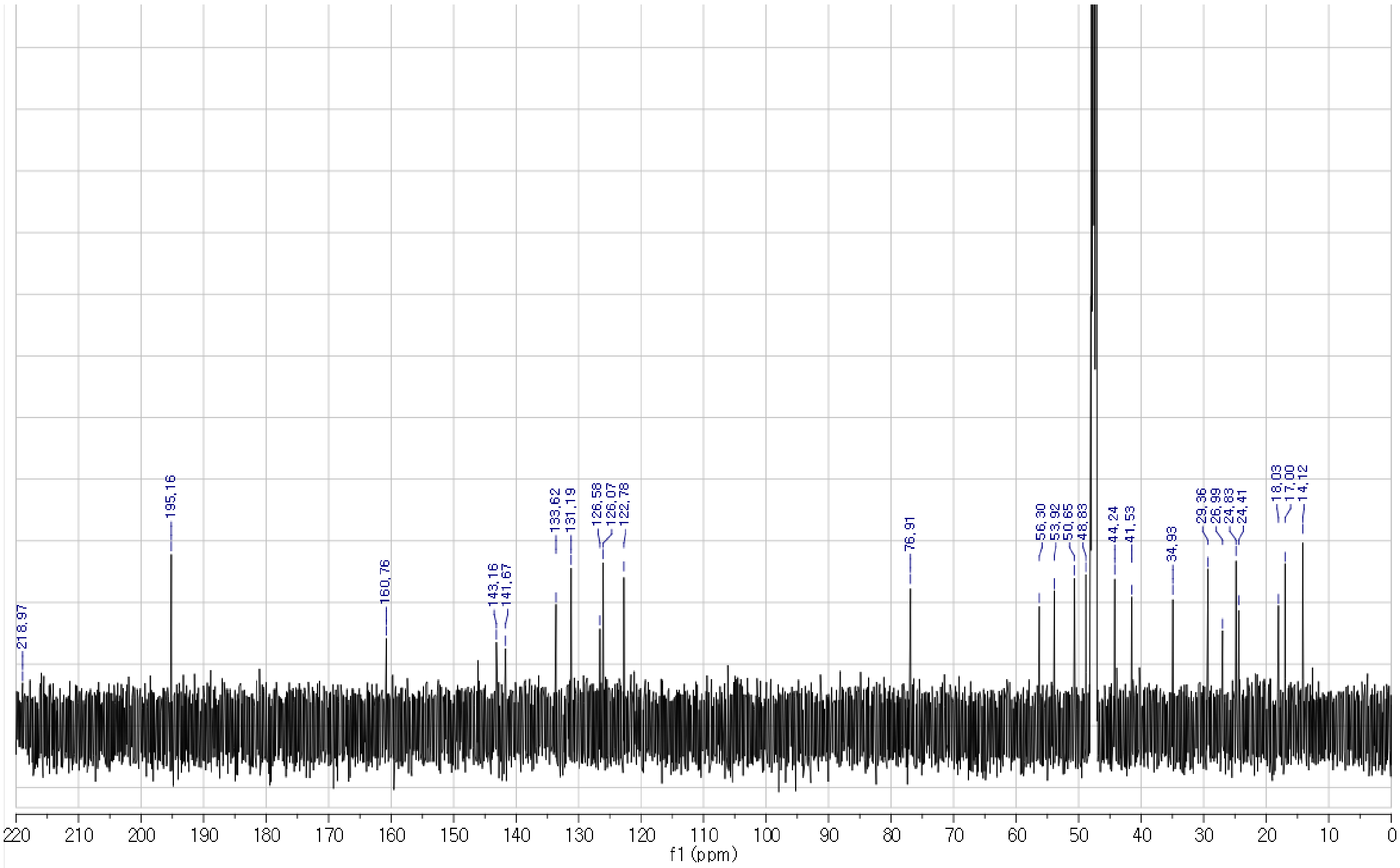

Figure S10. ^{13}C NMR spectrum of 14,15-dehydro-ophiobolin K (**2**).

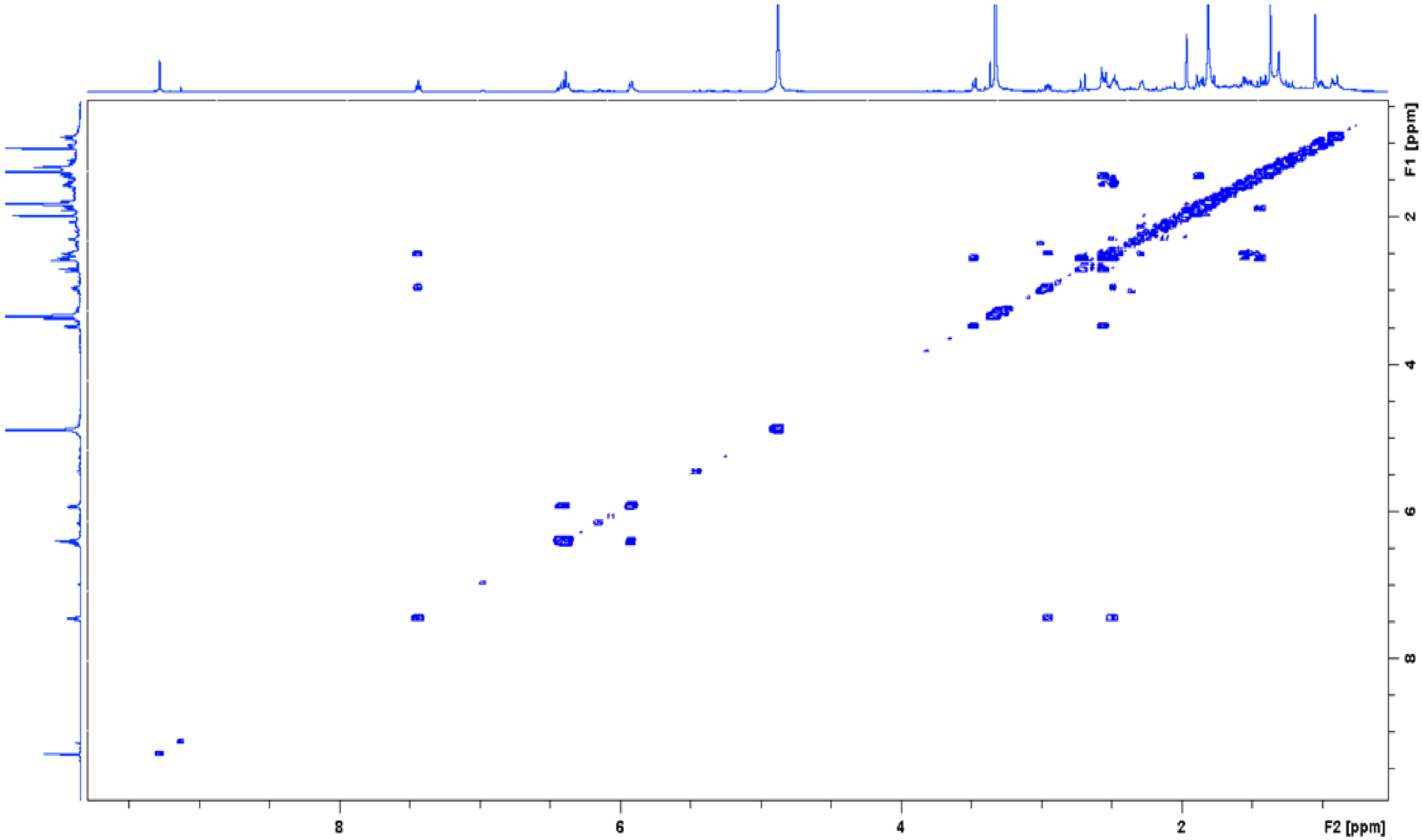

Figure S11. ^{1}H-^{1}H COSY spectrum of 14,15-dehydro-ophiobolin K (**2**).

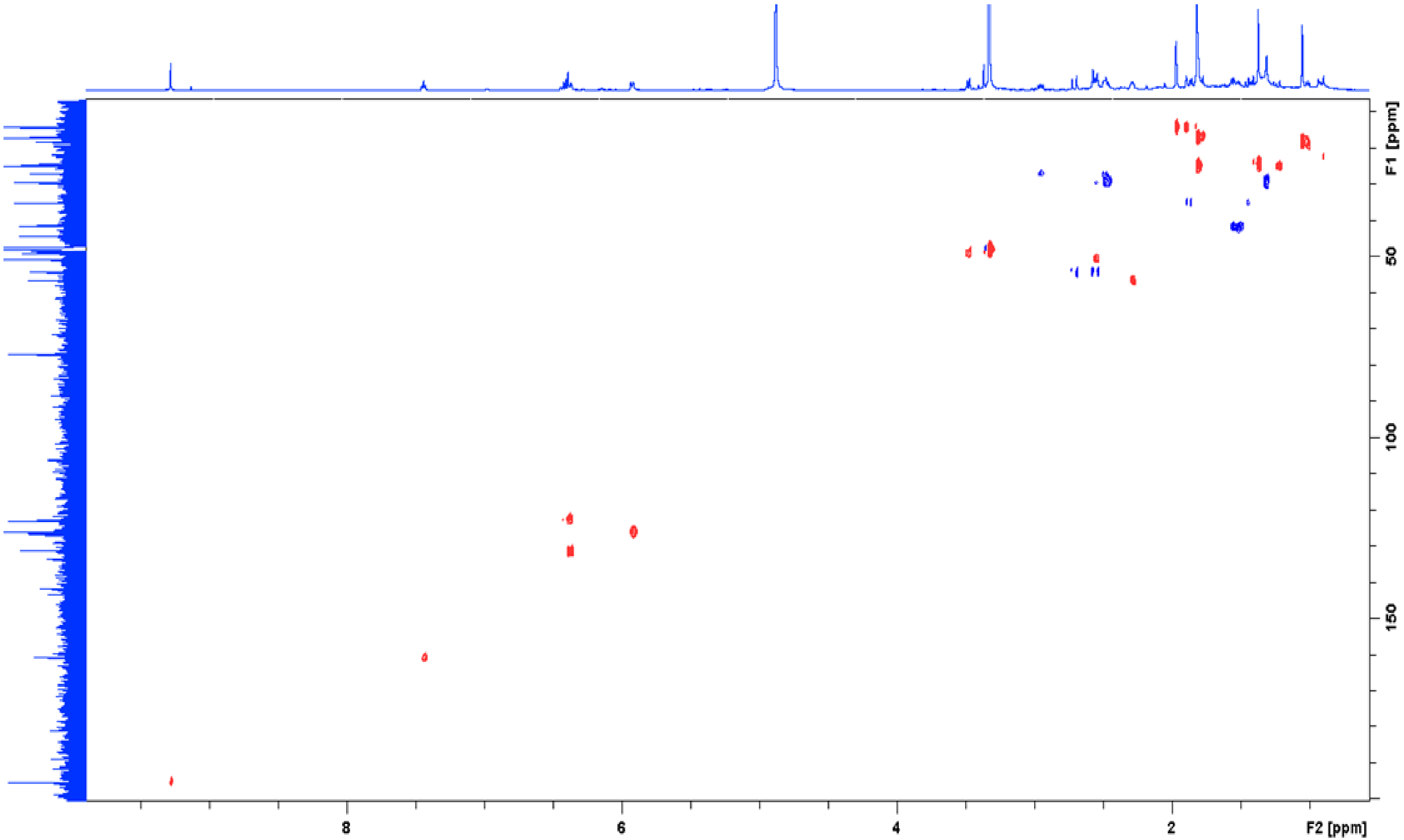

Figure S12. HSQC spectrum of 14,15-dehydro-ophiobolin K (**2**).

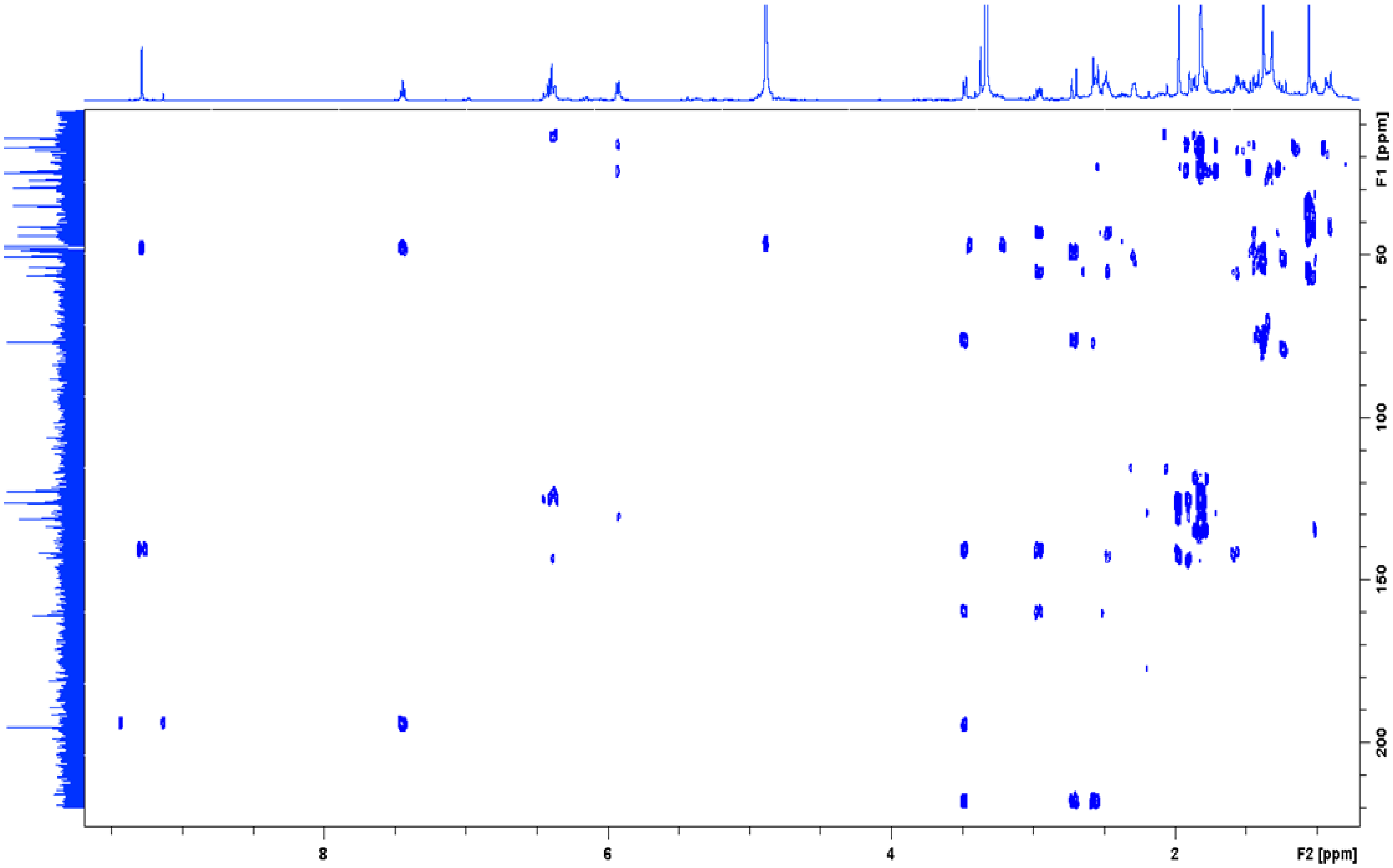

Figure S13. HMBC spectrum of 14,15-dehydro-ophiobolin K (**2**).

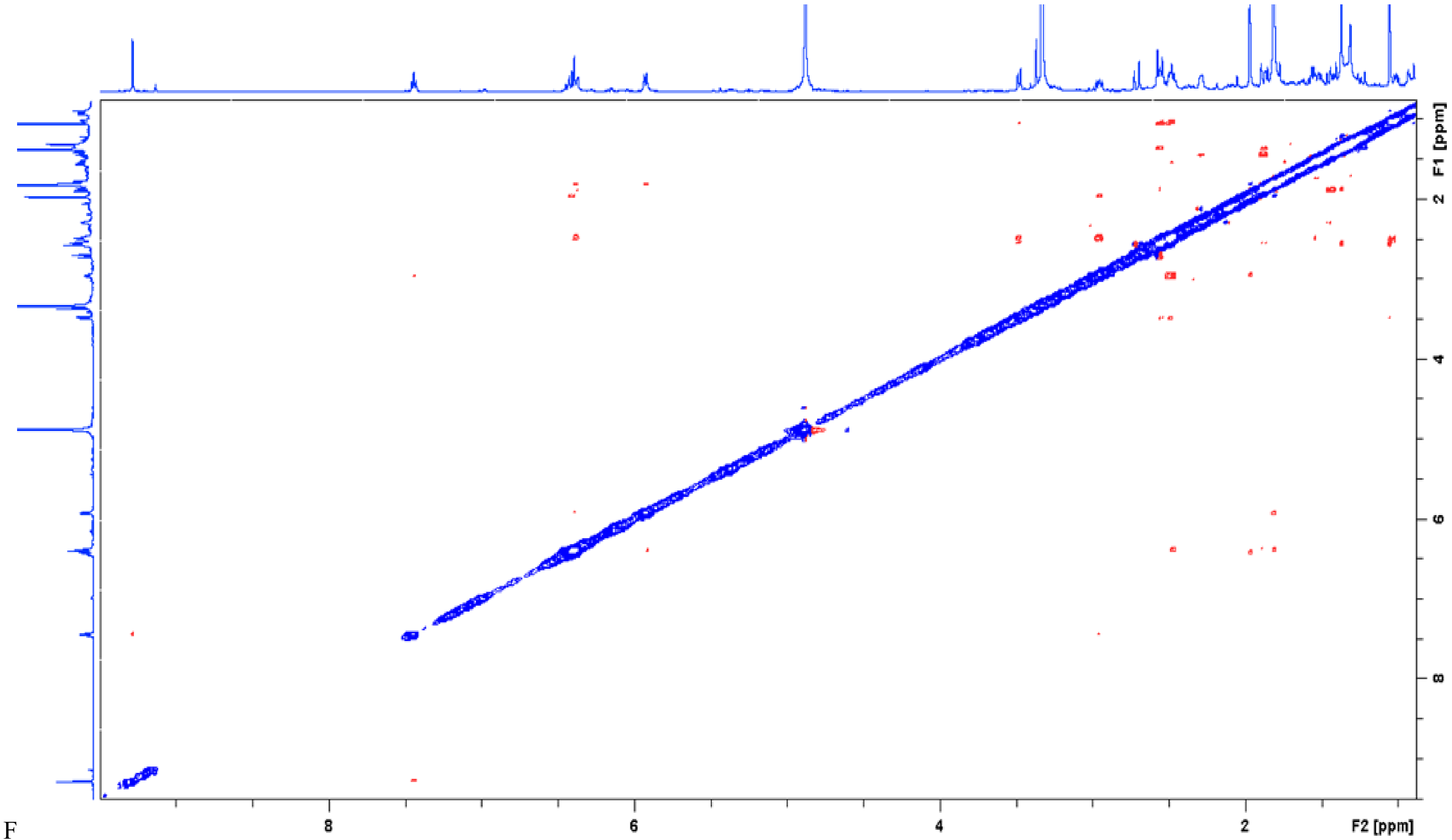

F

Figure S14. NOESY spectrum of 14,15-dehydro-ophiobolin K (**2**).

Elemental Composition Report

Single Mass Analysis
Tolerance = 5.0 PPM / DBE: min = -1.5, max = 50.0
Element prediction: Off
Number of isotope peaks used for i-FIT = 3

Monoisotopic Mass, Even Electron Ions
82 formula(e) evaluated with 1 results within limits (all results (up to 1000) for each mass)
Elements Used:
C: 1-55 H: 1-80 O: 1-10 Na: 0-1

Minimum:				-1.5				
Maximum:		500.0	5.0	50.0				
Mass	Calc. Mass	mDa	PPM	DBE	i-FIT	Norm	Conf(%)	Formula
387.2301	387.2300	0.1	0.3	9.5	1107.4	n/a	n/a	C25 H32 O2 Na

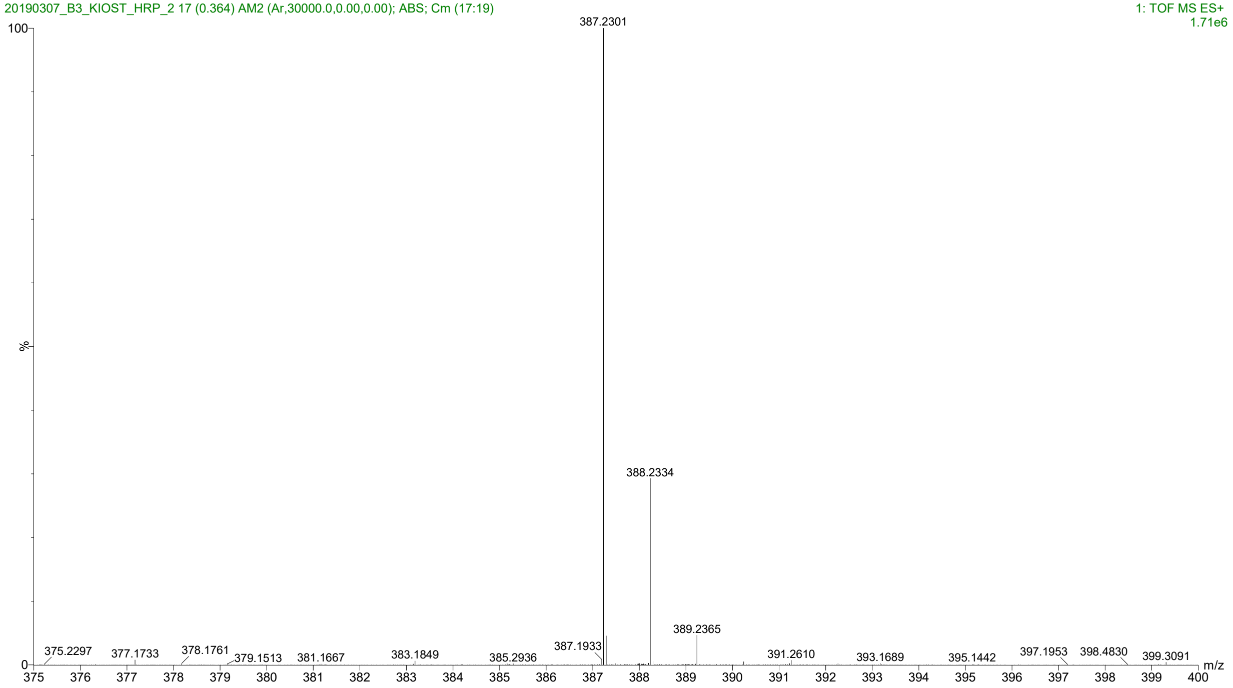

Figure S15. HRESIMS data of 14,15-dehydro-6-*epi*-ophiobolin G (**3**).

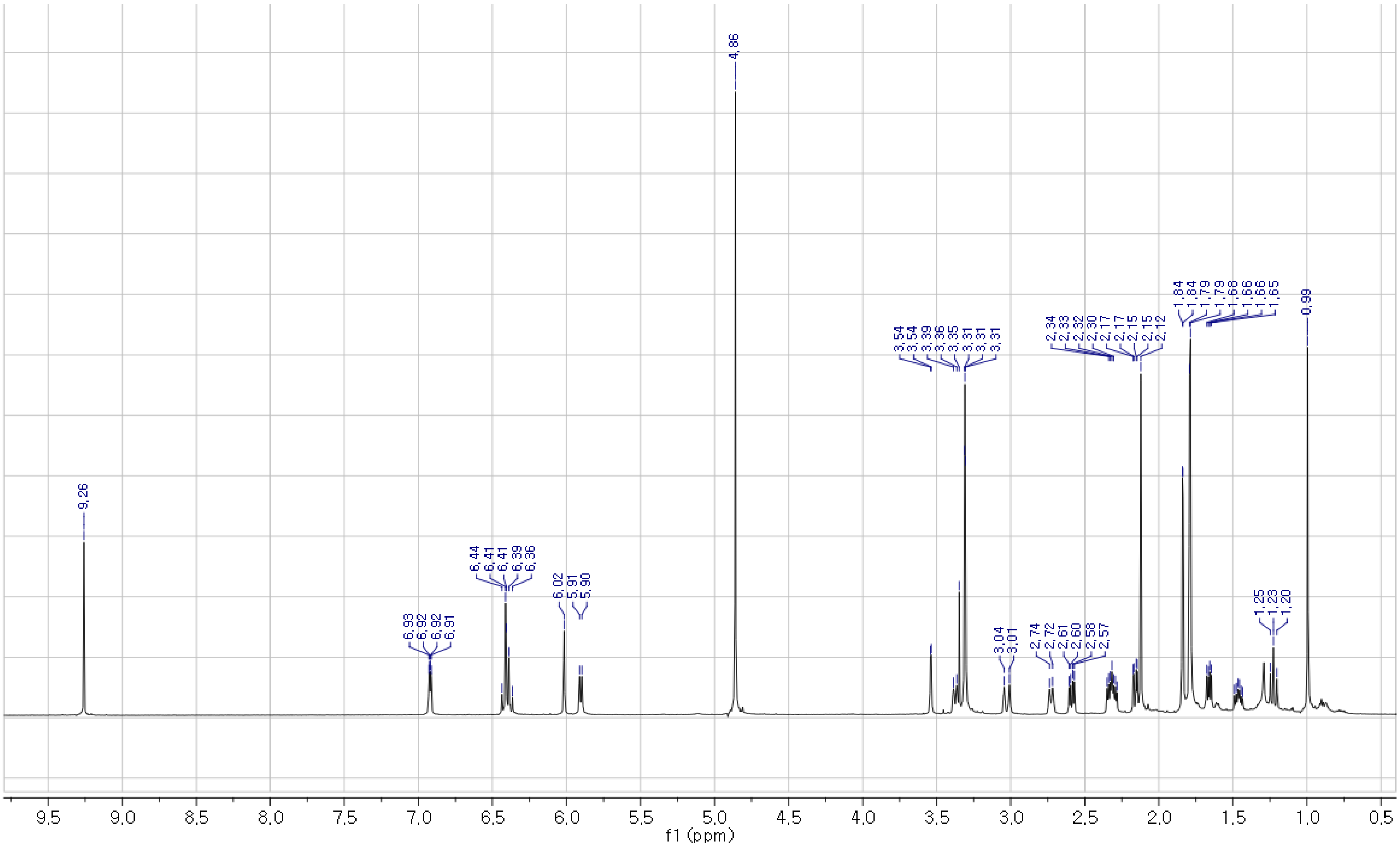

Figure S16. ^{1}H NMR spectrum of 14,15-dehydro-6-*epi*-ophiobolin G (**3**).

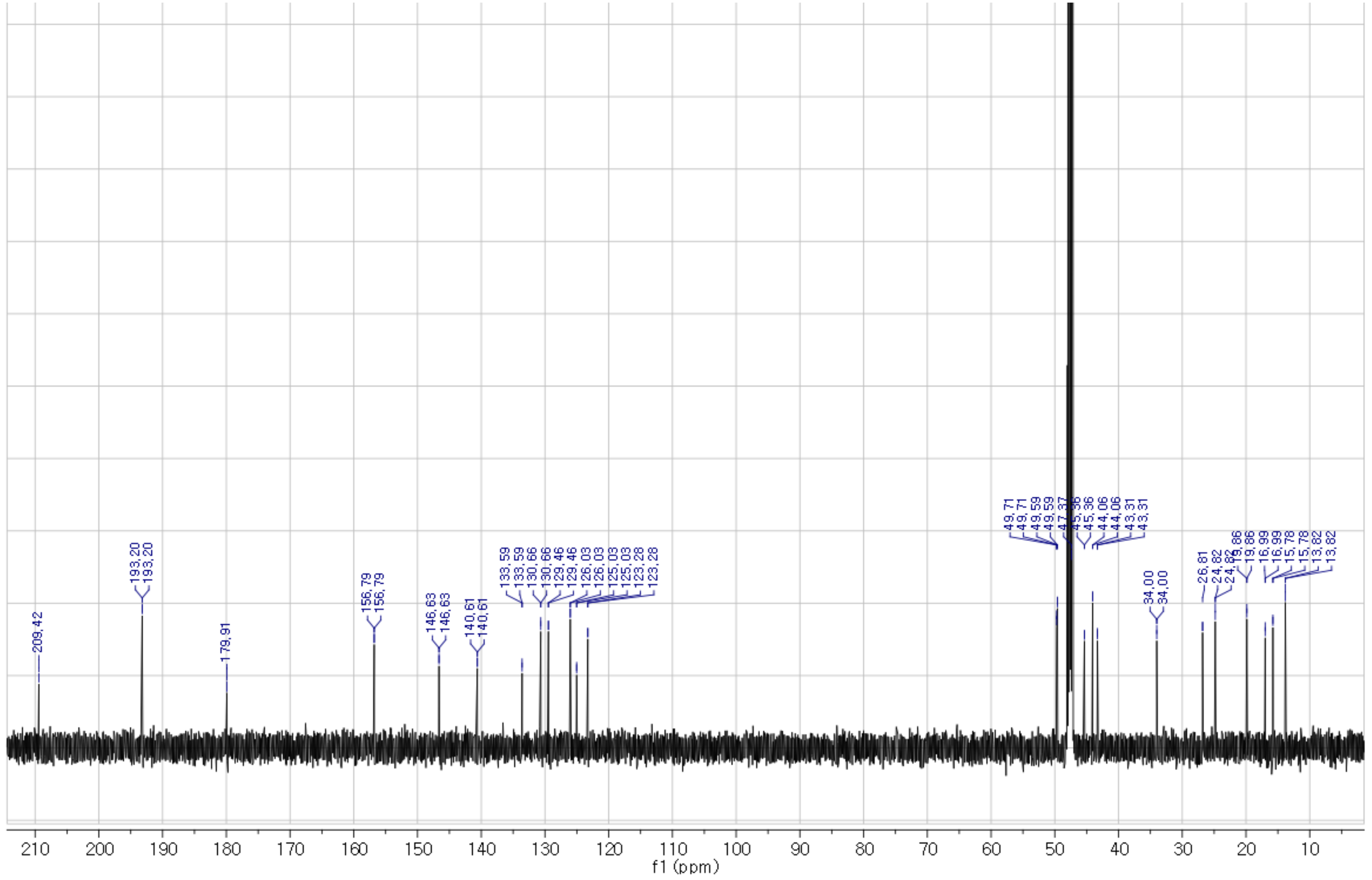

Figure S17. ^{13}C NMR spectrum of 14,15-dehydro-6-*epi*-ophiobolin G (**3**).

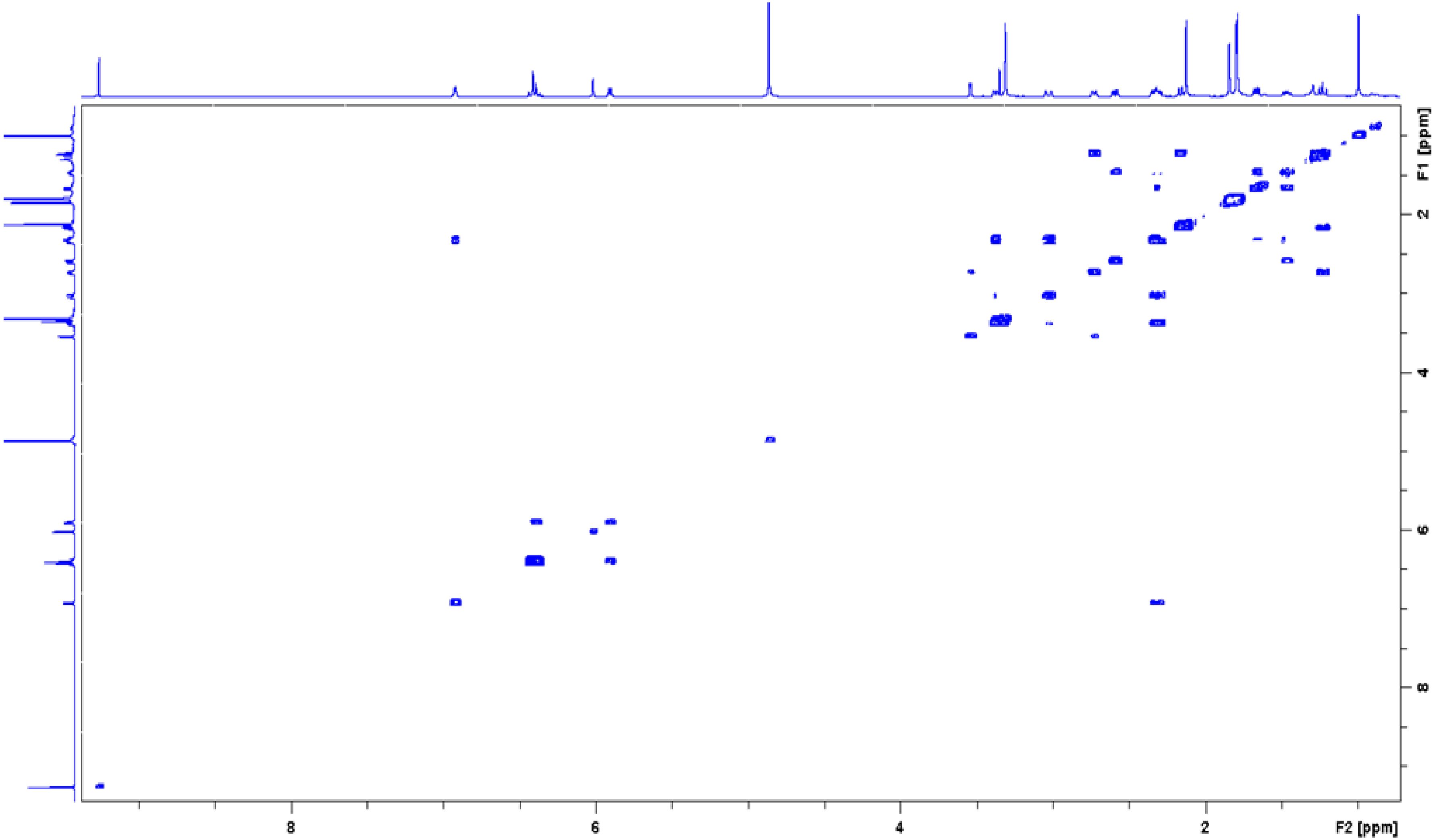

Figure S18. ^{1}H-^{1}H COSY spectrum of 14,15-dehydro-6-*epi*-ophiobolin G (**3**).

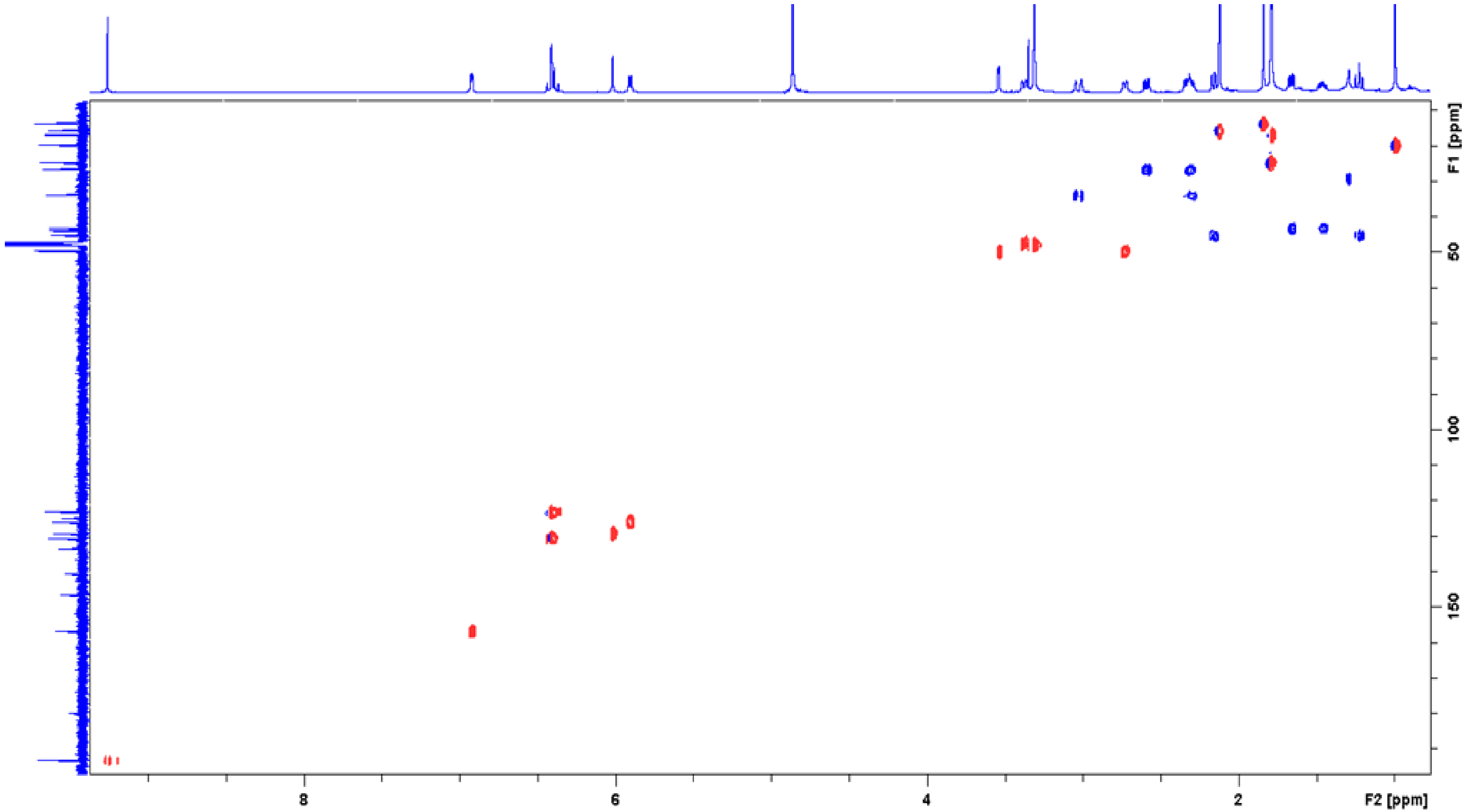

Figure S19. HSQC spectrum of 14,15-dehydro-6-*epi*-ophiobolin G (**3**).

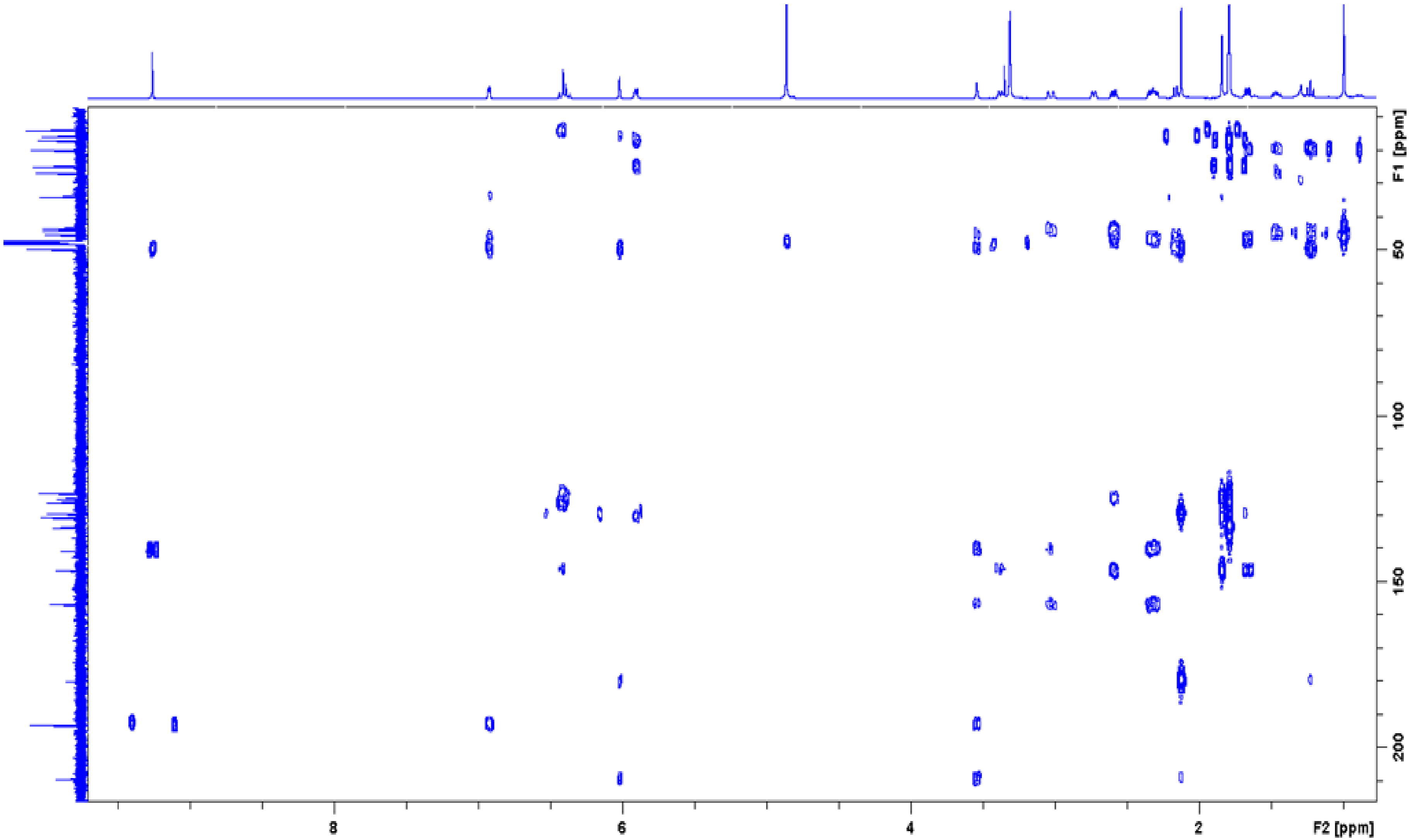

Figure S20. HMBC spectrum of 14,15-dehydro-6-*epi*-ophiobolin G (**3**).

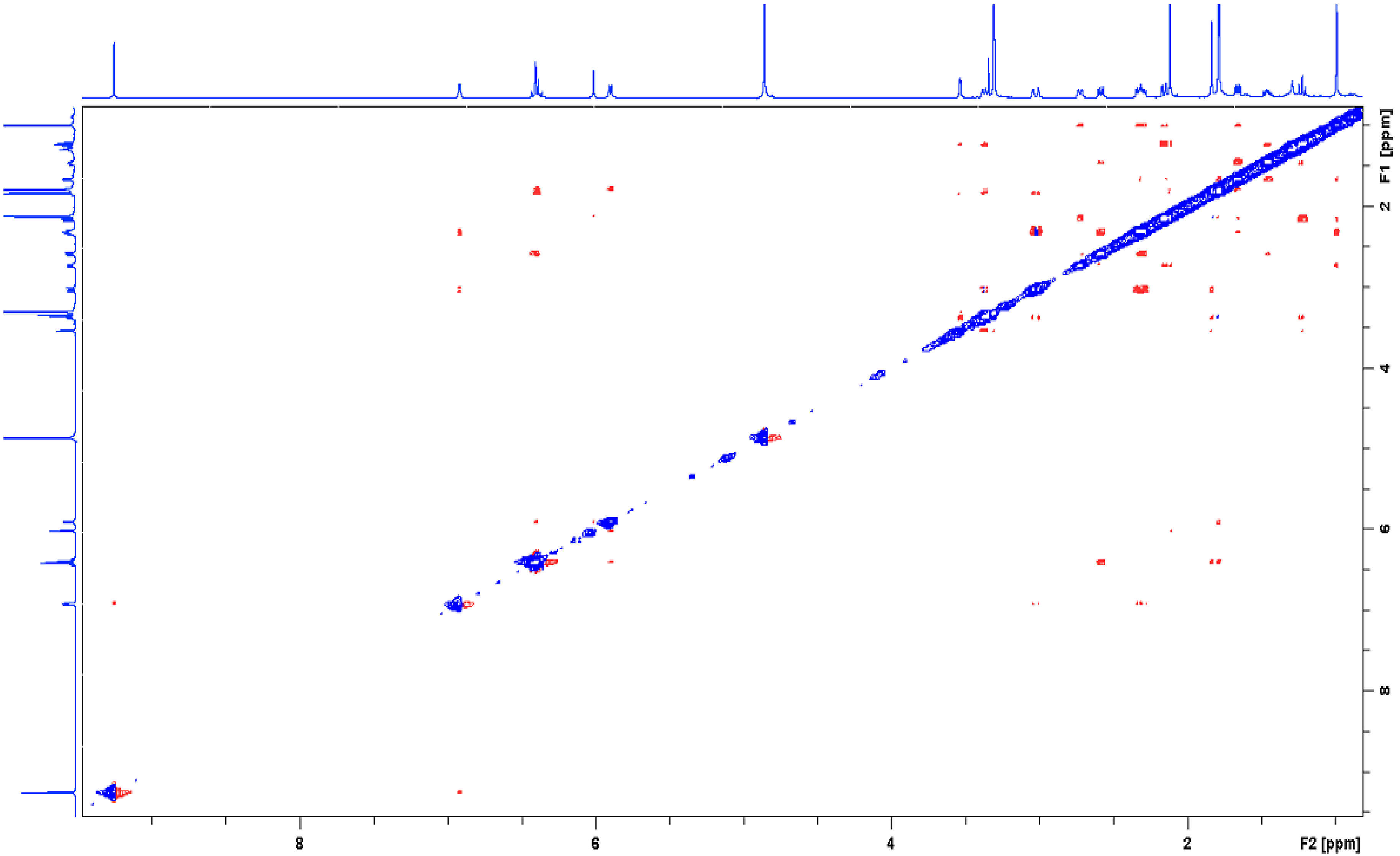

Figure S21.NOESY spectrum of 14,15-dehydro-6-*epi*-ophiobolin G (**3**).

Elemental Composition Report

Single Mass Analysis
Tolerance = 5.0 PPM / DBE: min = -1.5, max = 50.0
Element prediction: Off
Number of isotope peaks used for i-FIT = 3

Monoisotopic Mass, Even Electron Ions
40 formula(e) evaluated with 1 results within limits (all results (up to 1000) for each mass)
Elements Used:
C: 1-30 H: 1-50 O: 1-5 Na: 0-1

Minimum:				-1.5				
Maximum:		500.0	5.0	50.0				
Mass	Calc. Mass	mDa	PPM	DBE	i-FIT	Norm	Conf(%)	Formula
387.2299	387.2300	-0.1	-0.3	9.5	1248.9	n/a	n/a	C25 H32 O2 Na

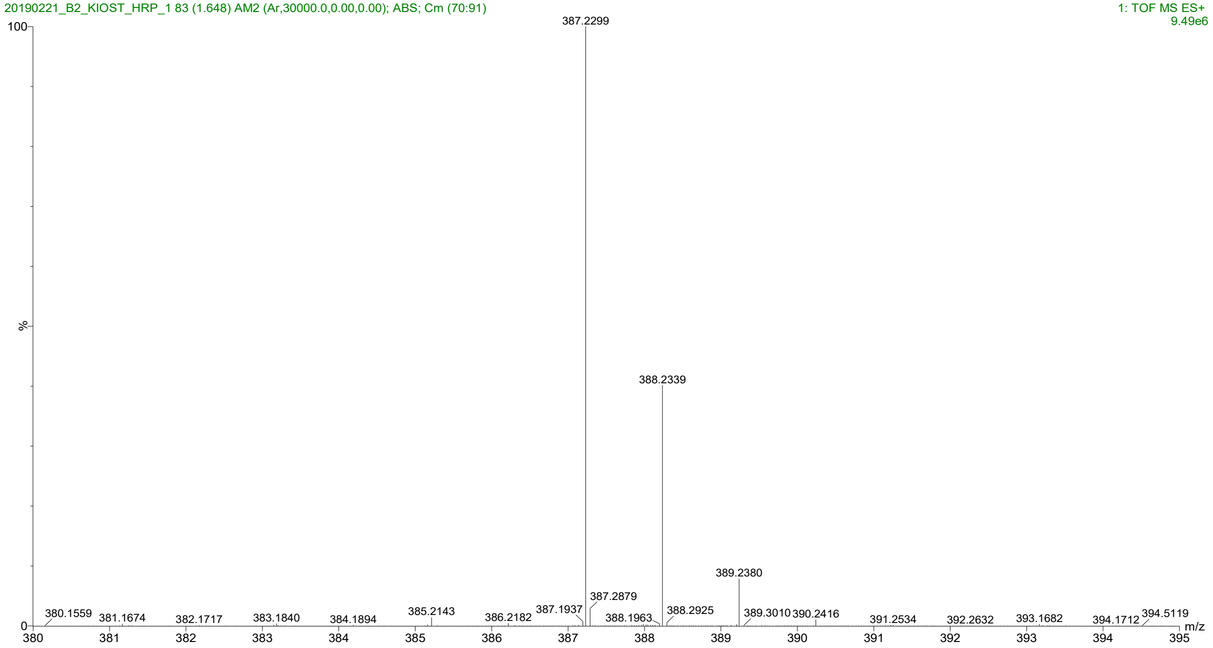

Figure S22. HRESIMS data of 14,15-dehydro-ophiobolin G (**4**).

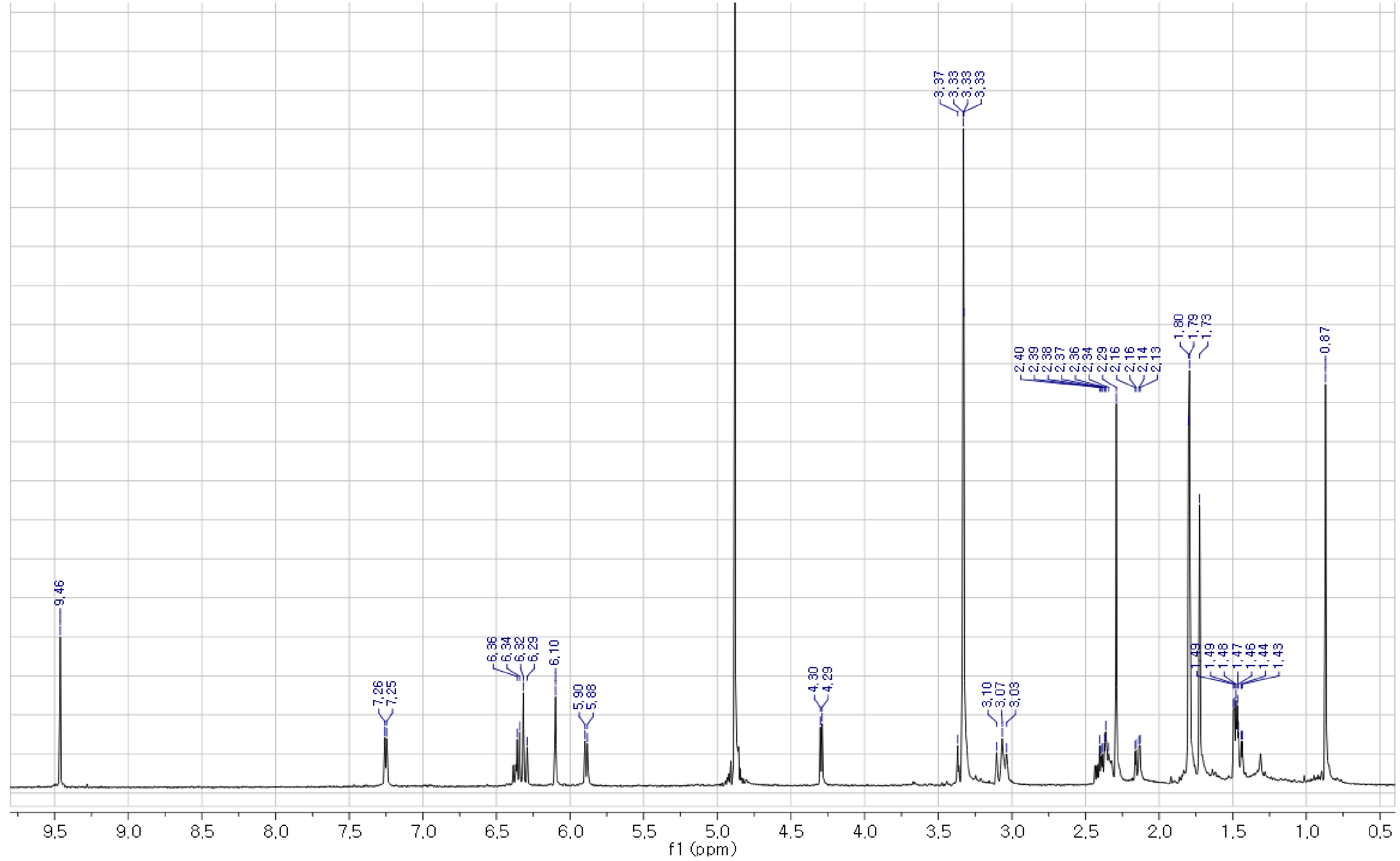

Figure S23. ^{1}H NMR spectrum of 14,15-dehydro-ophiobolin G (**4**).

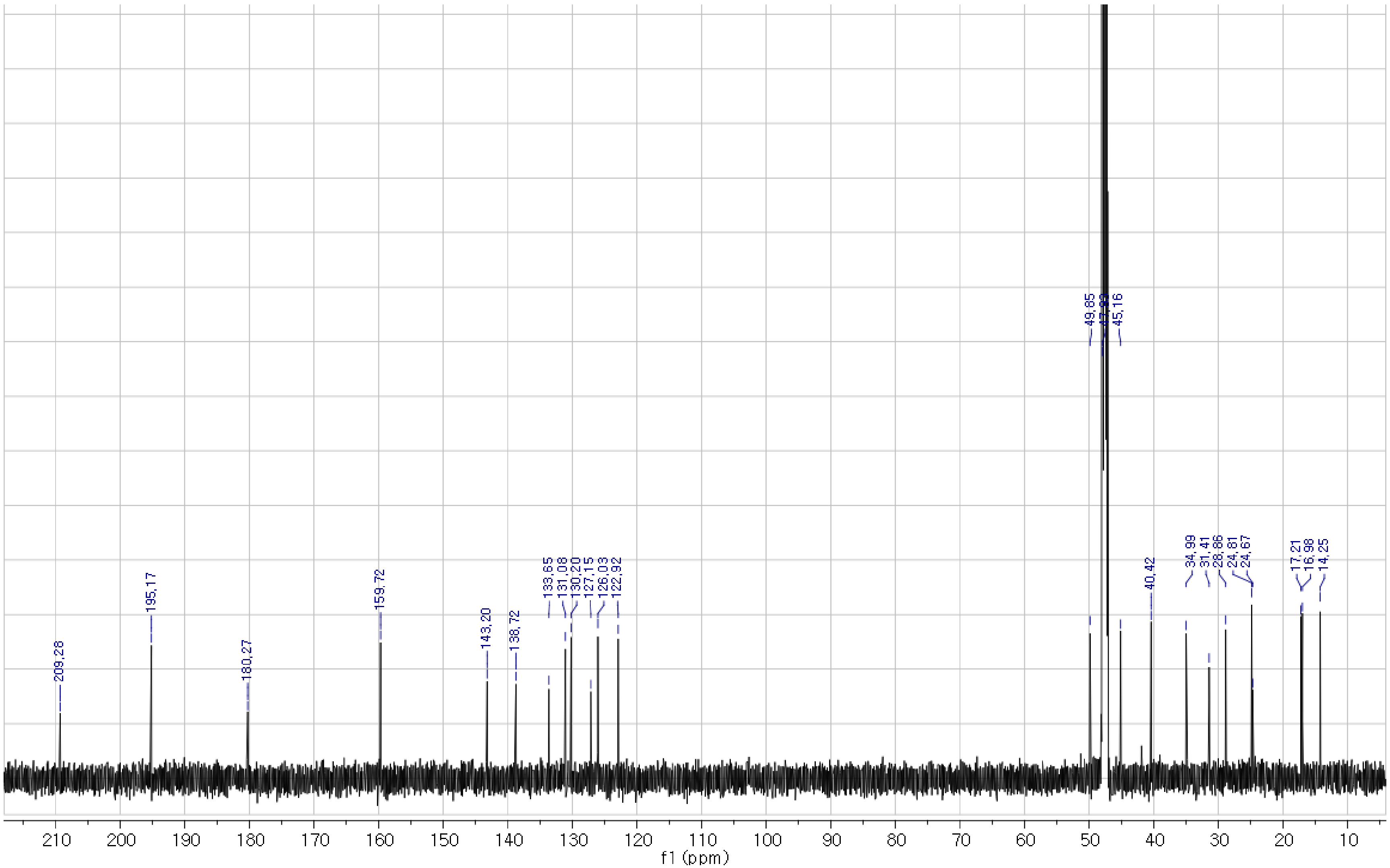

Figure S24. ^{13}C NMR spectrum of 14,15-dehydro-ophiobolin G (**4**).

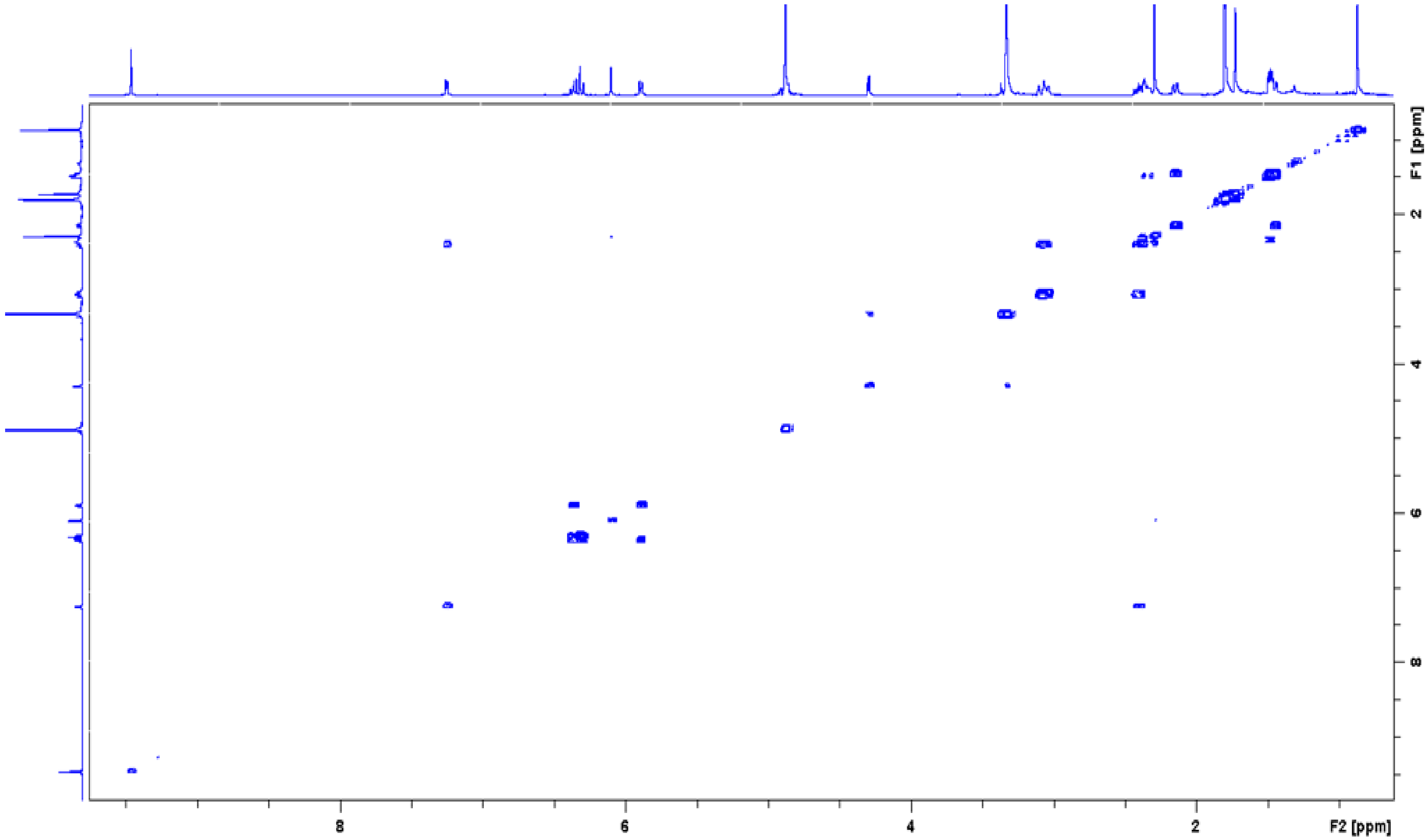

Figure S25. ^{1}H-^{1}H COSY spectrum of 14,15-dehydro-ophiobolin G (**4**).

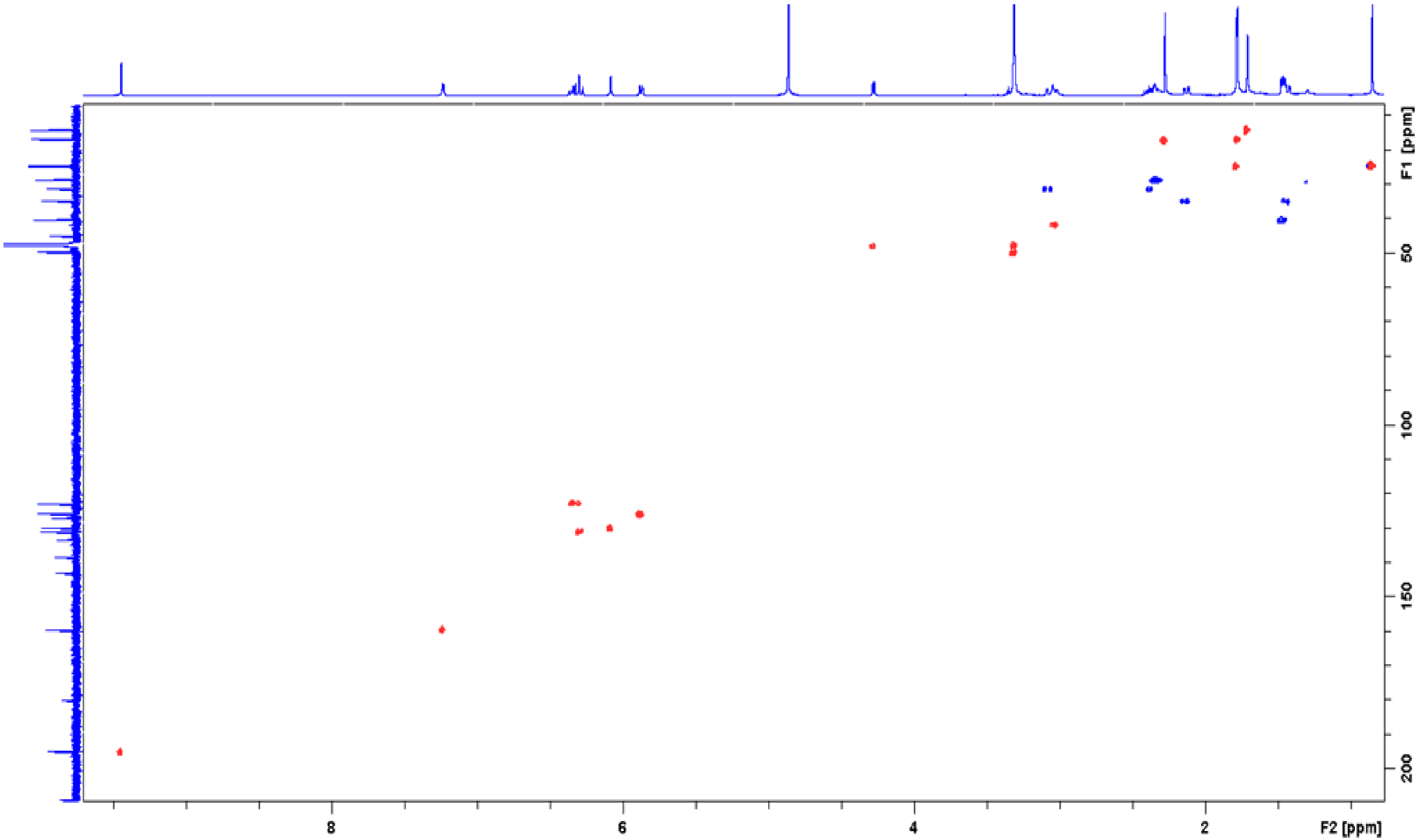

Figure S26. HSQC spectrum of 14,15-dehydro-ophiobolin G (**4**).

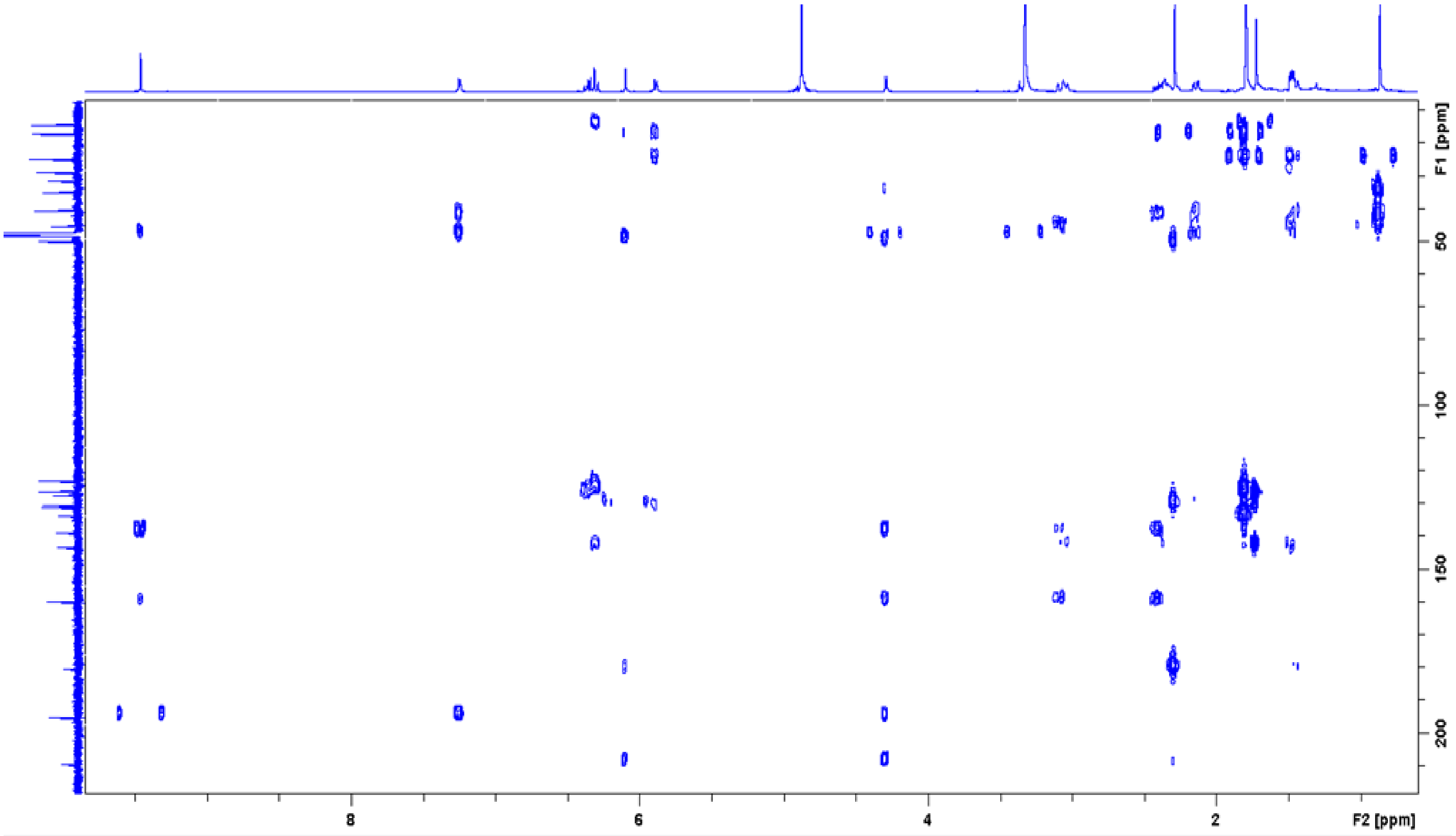

Figure S27. HMBC spectrum of 14,15-dehydro-ophiobolin G (**4**).

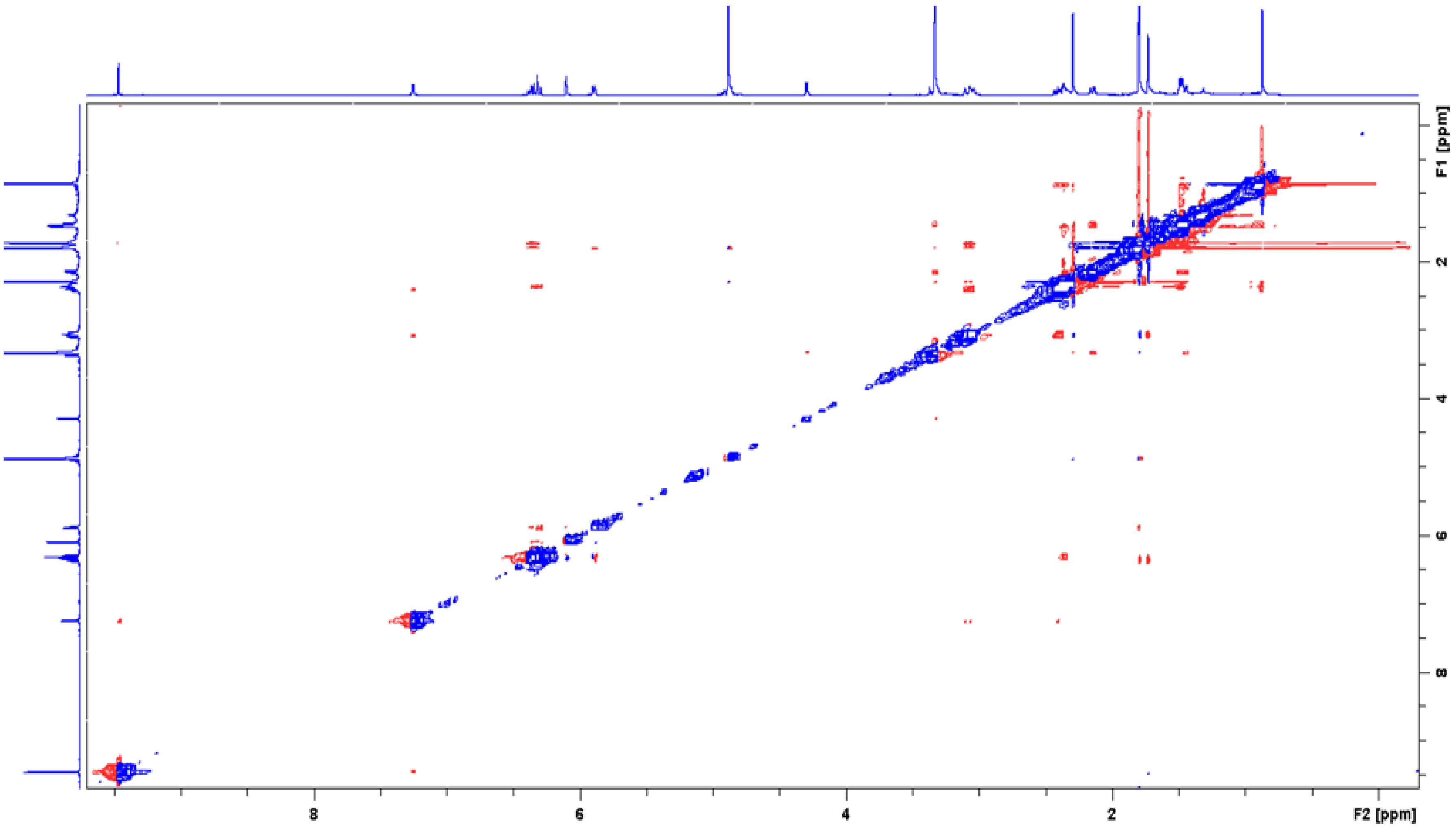

Figure S28. NOESY spectrum of 14,15-dehydro-ophiobolin G (**4**).

Elemental Composition Report

Single Mass Analysis
Tolerance = 5.0 PPM / DBE: min = -1.5, max = 50.0
Element prediction: Off
Number of isotope peaks used for i-FIT = 3

Monoisotopic Mass, Even Electron Ions
82 formula(e) evaluated with 1 results within limits (all results (up to 1000) for each mass)
Elements Used:
C: 1-55 H: 1-80 O: 1-10 Na: 0-1

Minimum:				-1.5				
Maximum:		500.0	5.0	50.0				
Mass	Calc. Mass	mDa	PPM	DBE	i-FIT	Norm	Conf(%)	Formula
387.2299	387.2300	-0.1	-0.3	9.5	1135.1	n/a	n/a	C25 H32 O2 Na

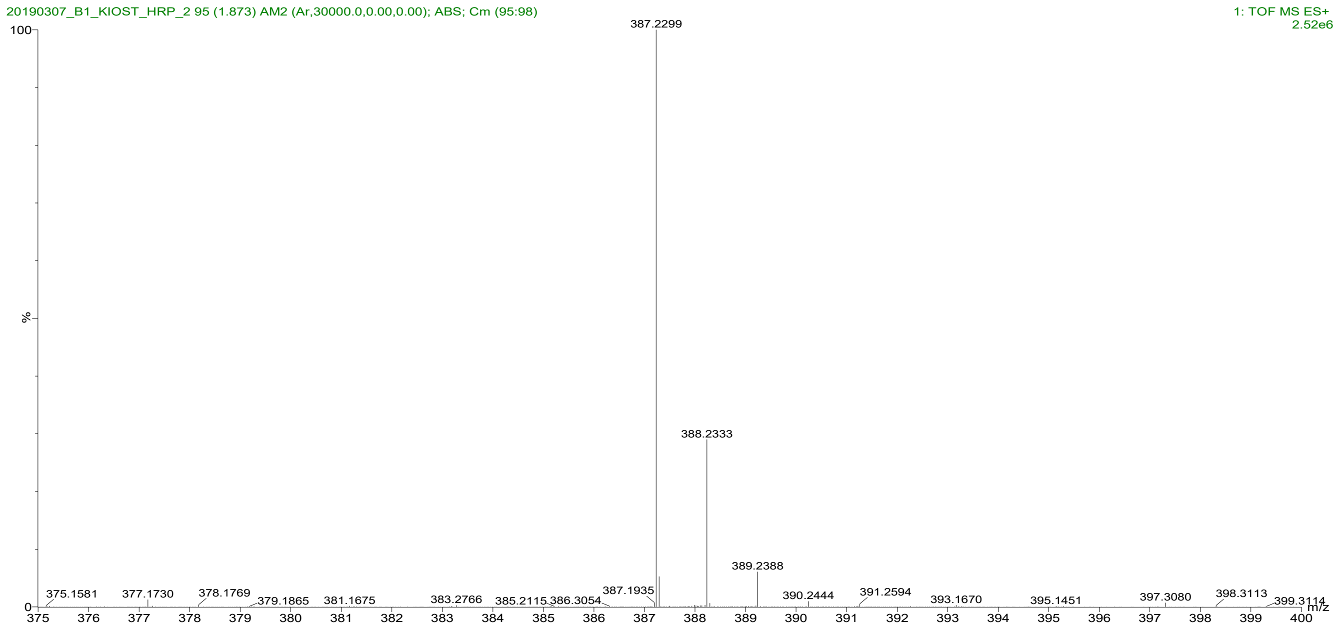

Figure S29. HRESIMS data of 14,15-dehydro-(*Z*)-14-ophiobolin G (**5**).

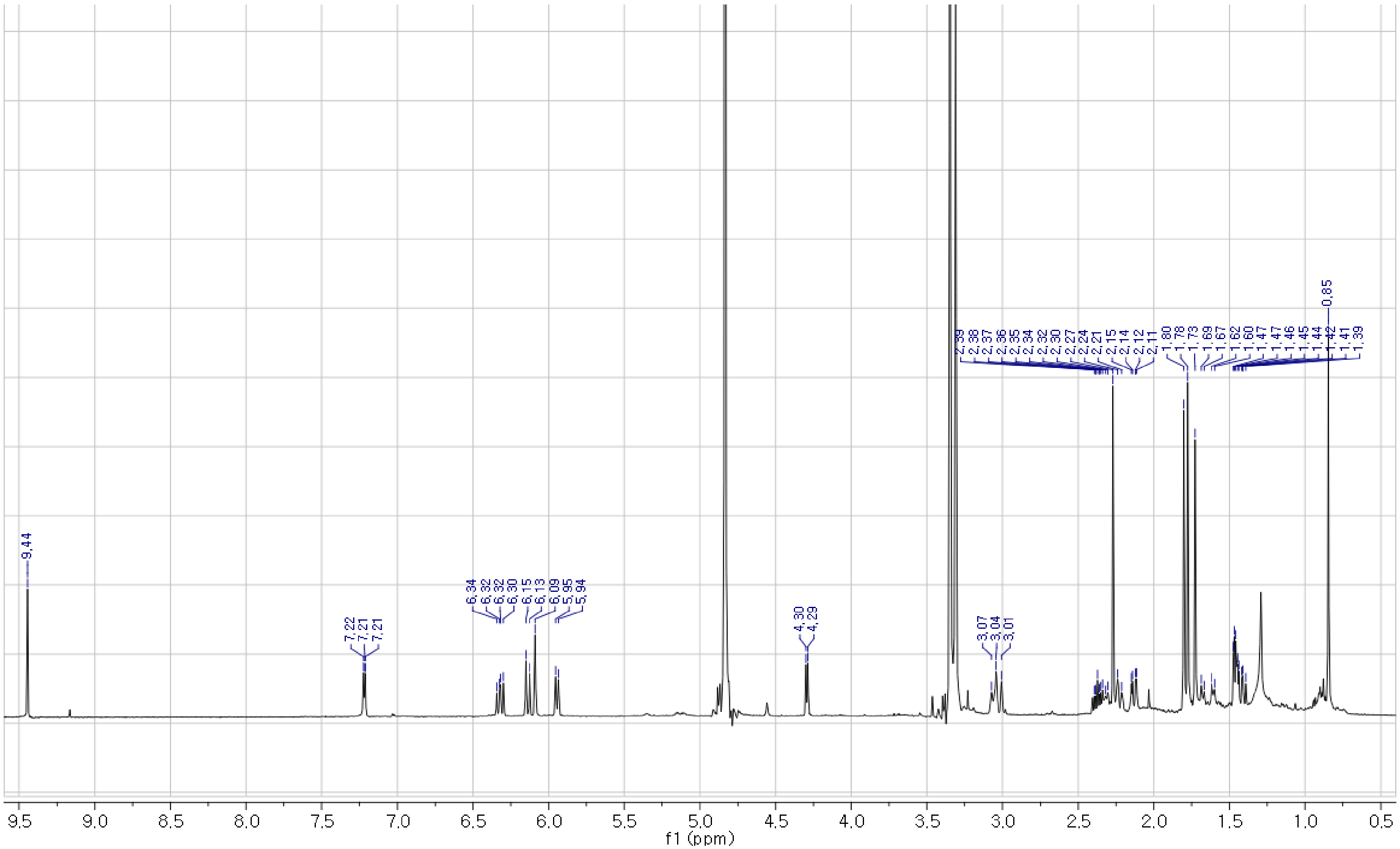

Figure S30. ^{1}H NMR spectrum of 14,15-dehydro-(*Z*)-14-ophiobolin G (**5**).

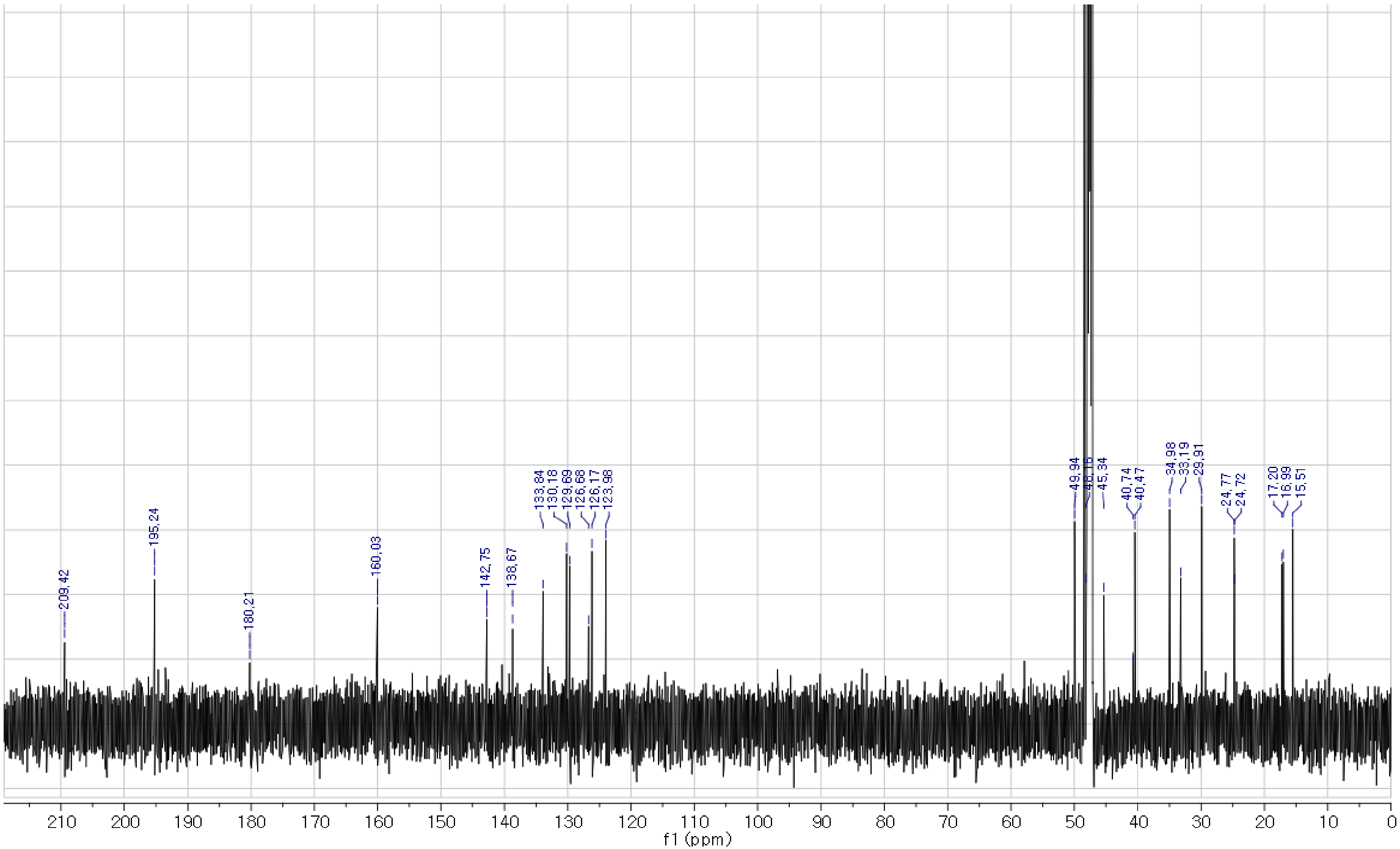

Figure S31. ^{13}C NMR spectrum of 14,15-dehydro-(*Z*)-14-ophiobolin G (**5**).

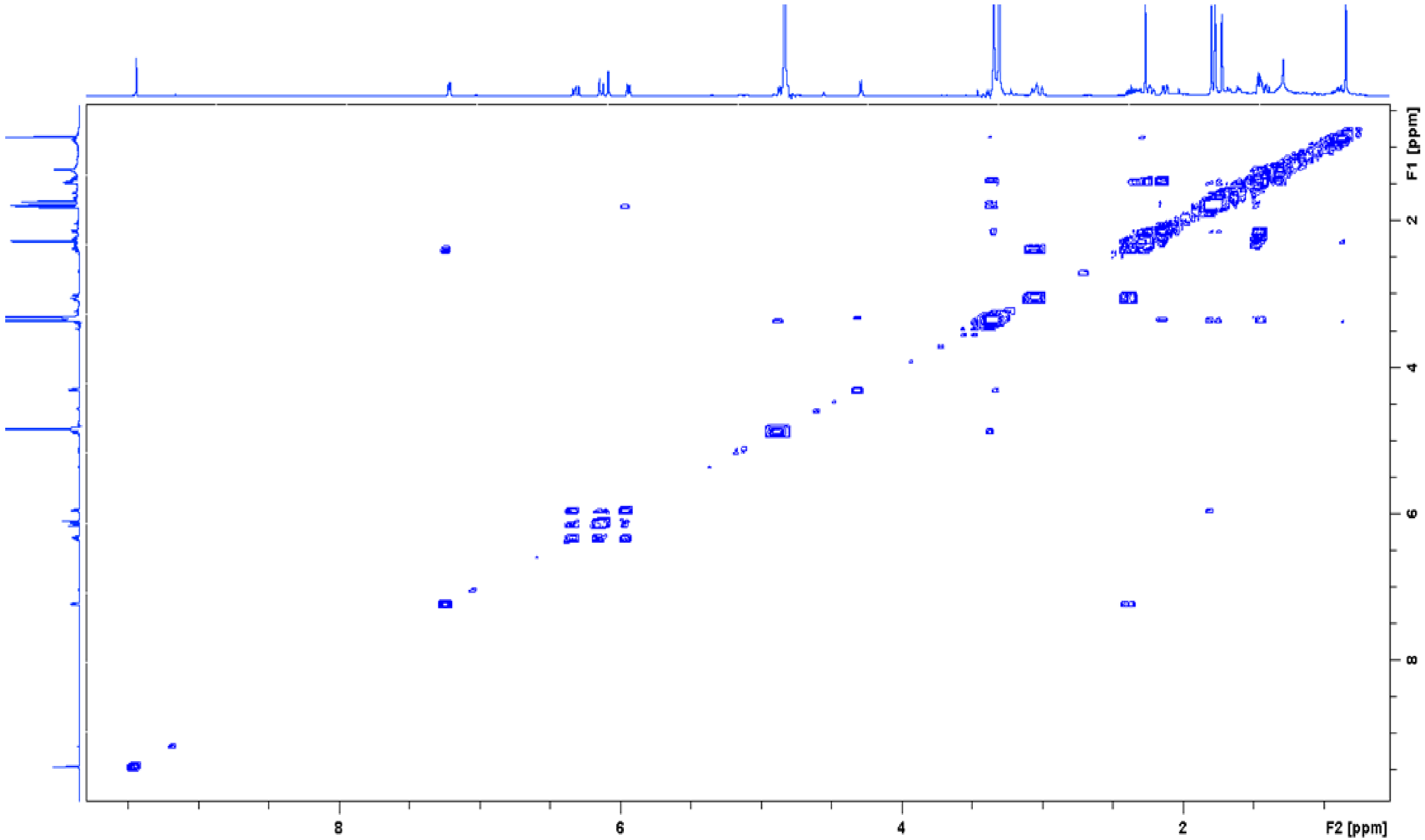

Figure S32. ^{1}H-^{1}H COSY spectrum of 14,15-dehydro-(*Z*)-14-ophiobolin G (**5**).

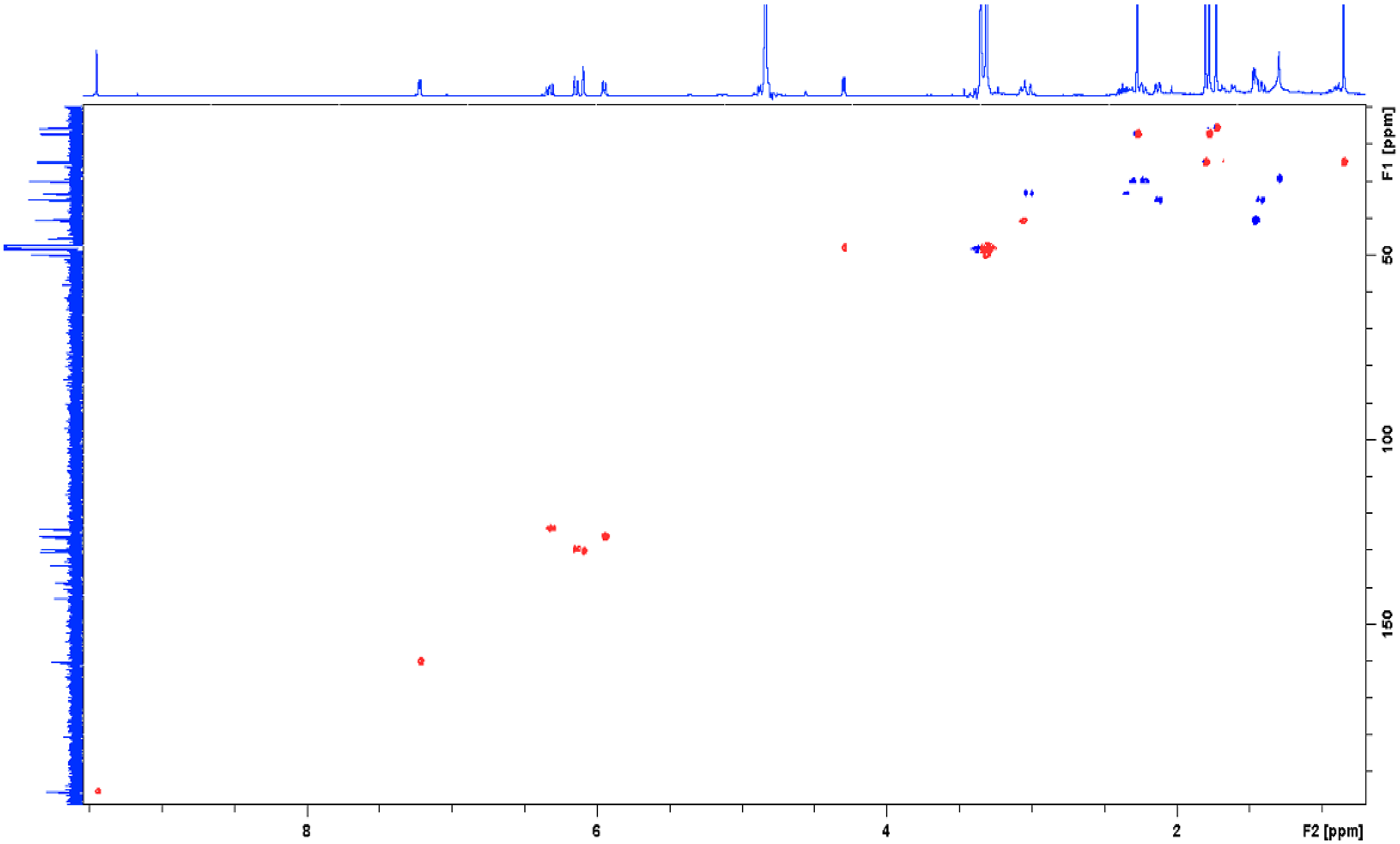

Figure S33. HSQC spectrum of 14,15-dehydro-(*Z*)-14-ophiobolin G (**5**).

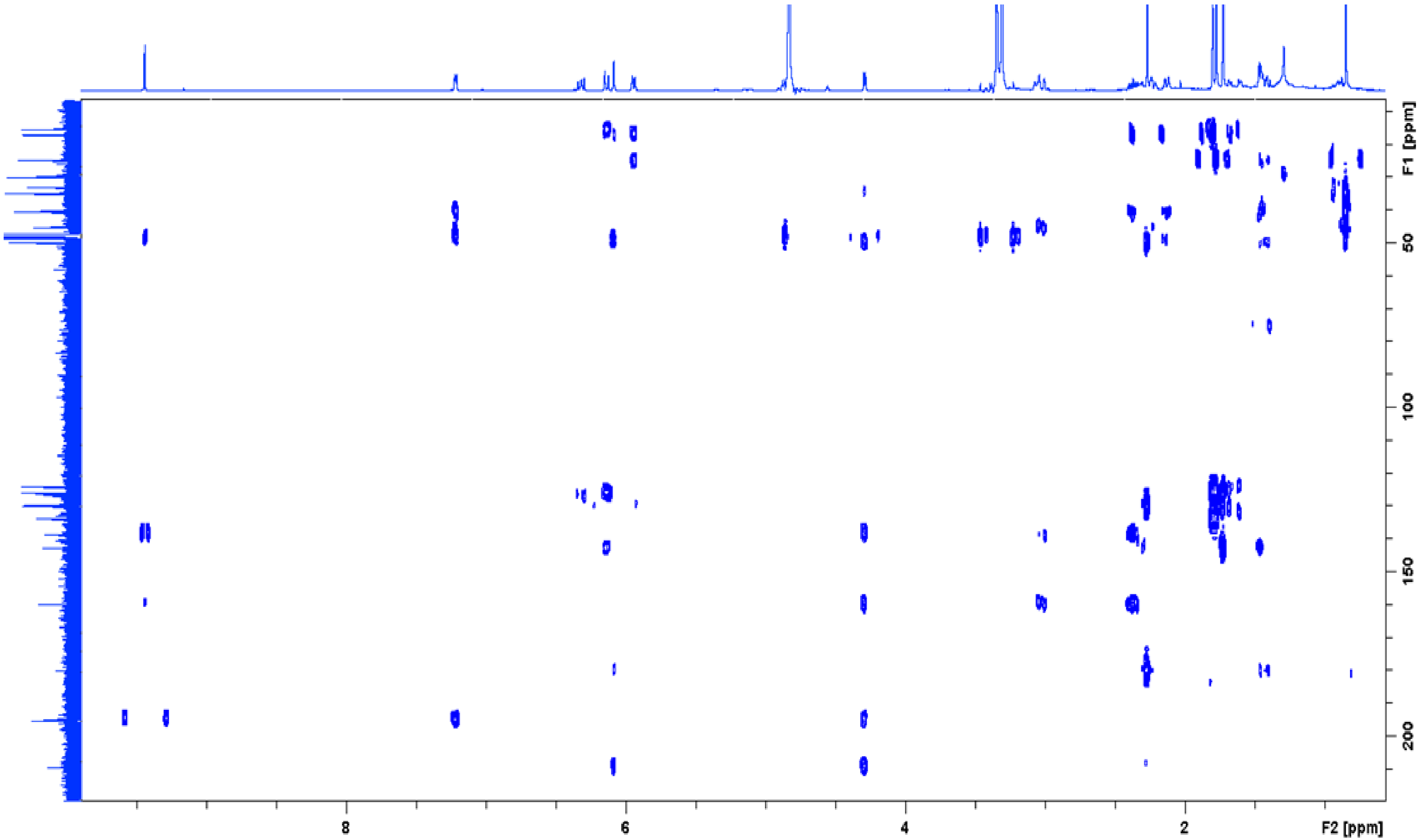

Figure S34. HMBC spectrum of 14,15-dehydro-(*Z*)-14-ophiobolin G (**5**).

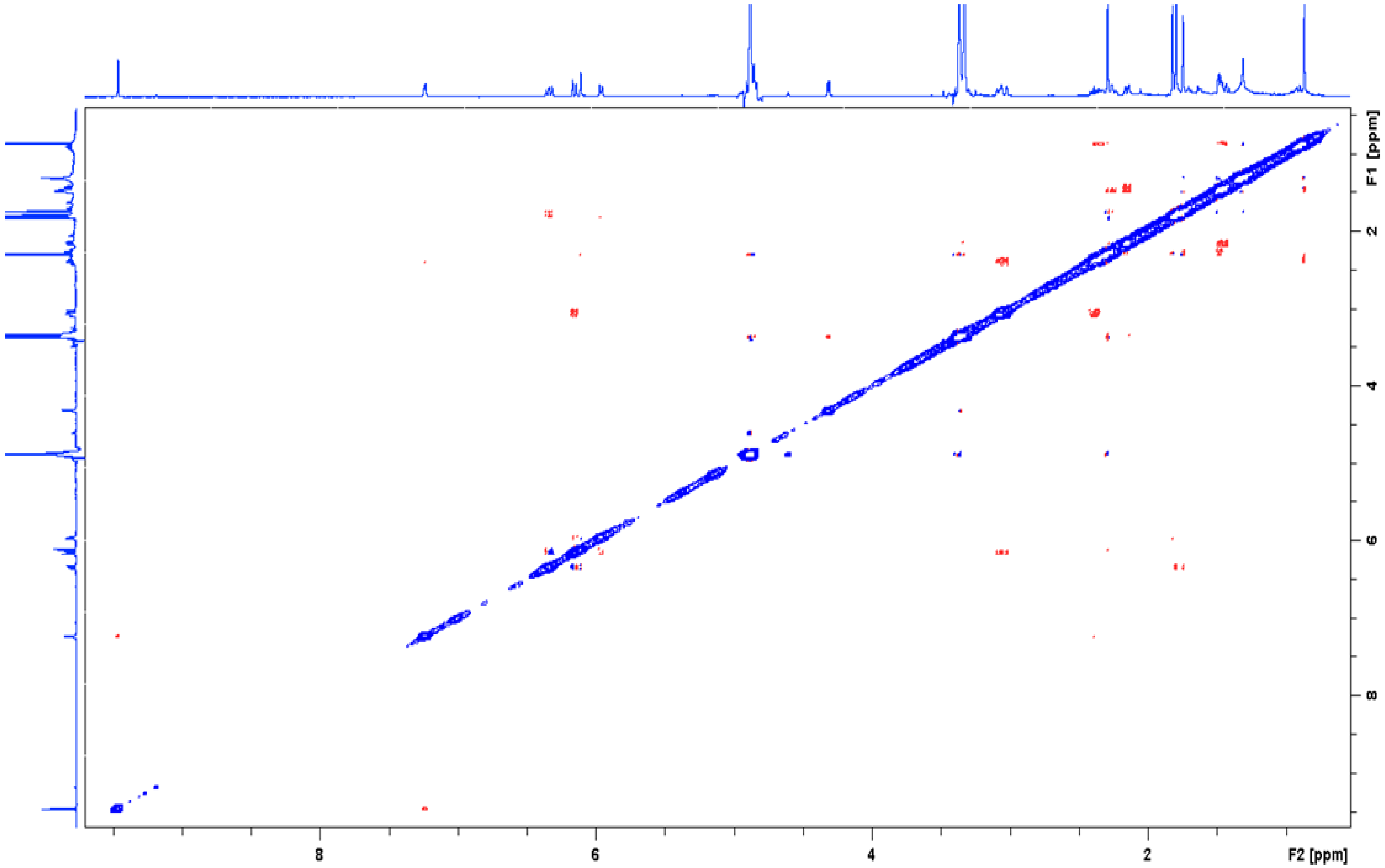

Figure S35. NOESY spectrum of 14,15-dehydro-(*Z*)-14-ophiobolin G (**5**).

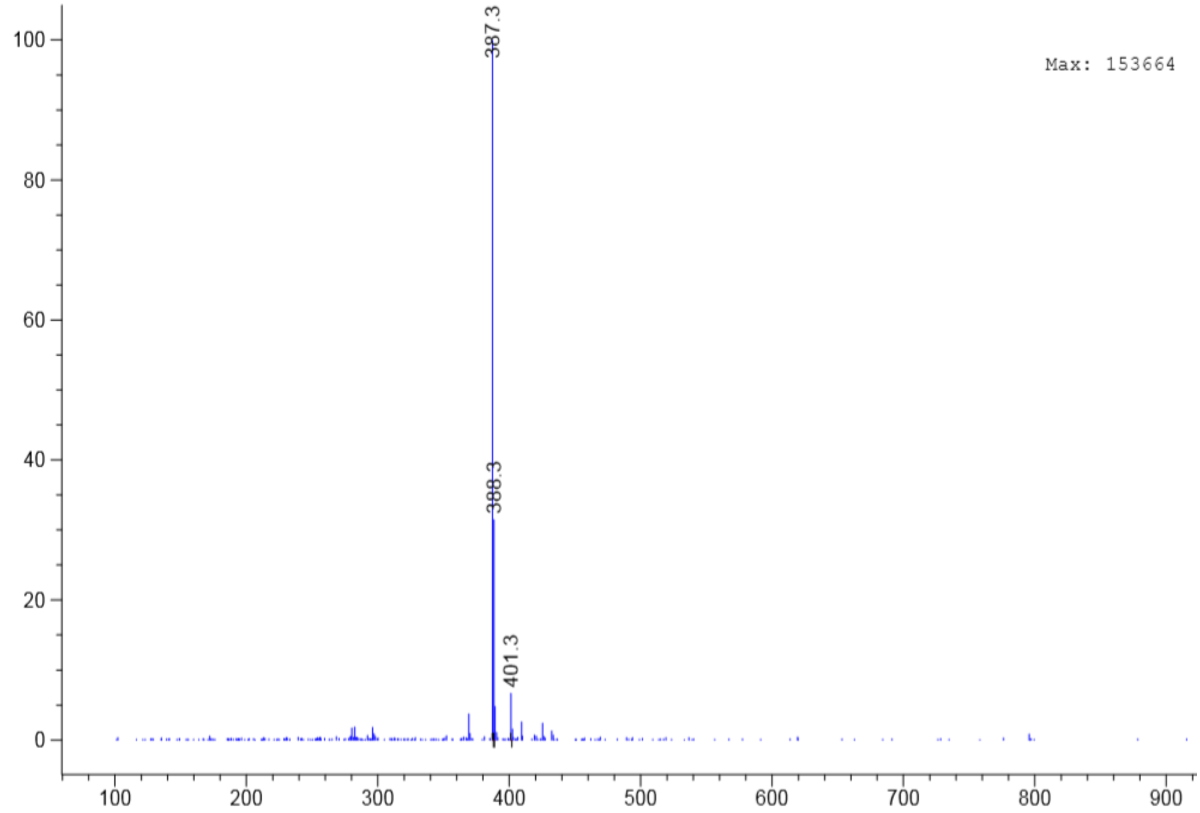

Figure S36. LRMS data of 6-*epi*-ophiobolin C (**6**).

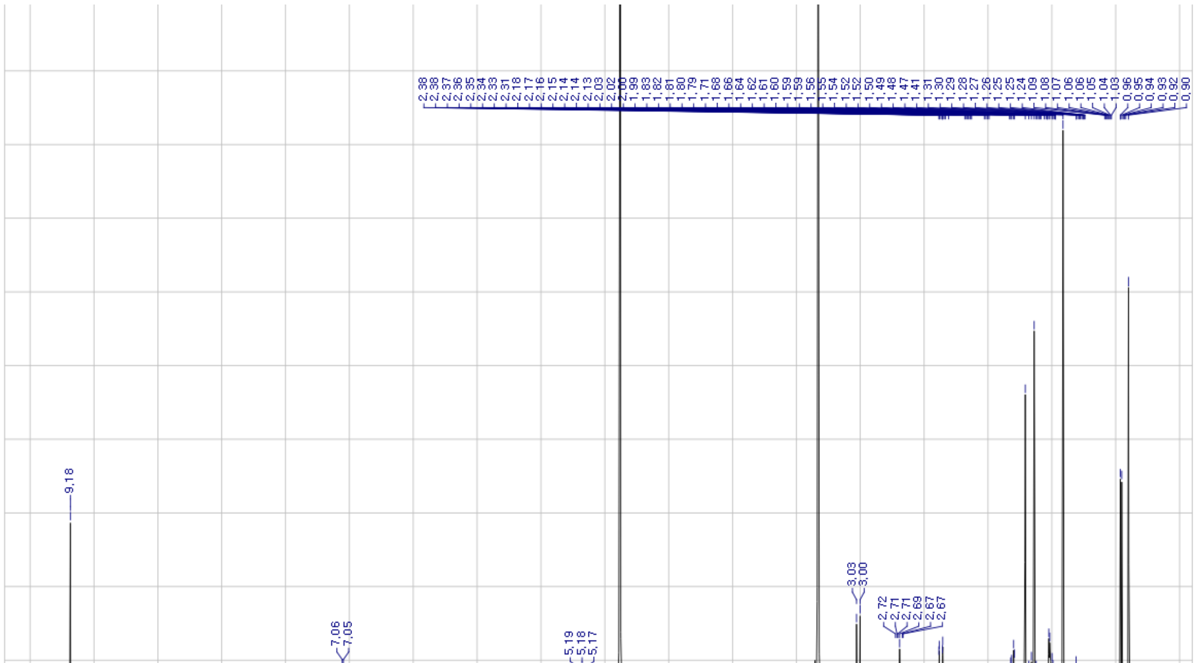

Figure S37. ^{1}H NMR spectrum of 6-*epi*-ophiobolin C (**6**).

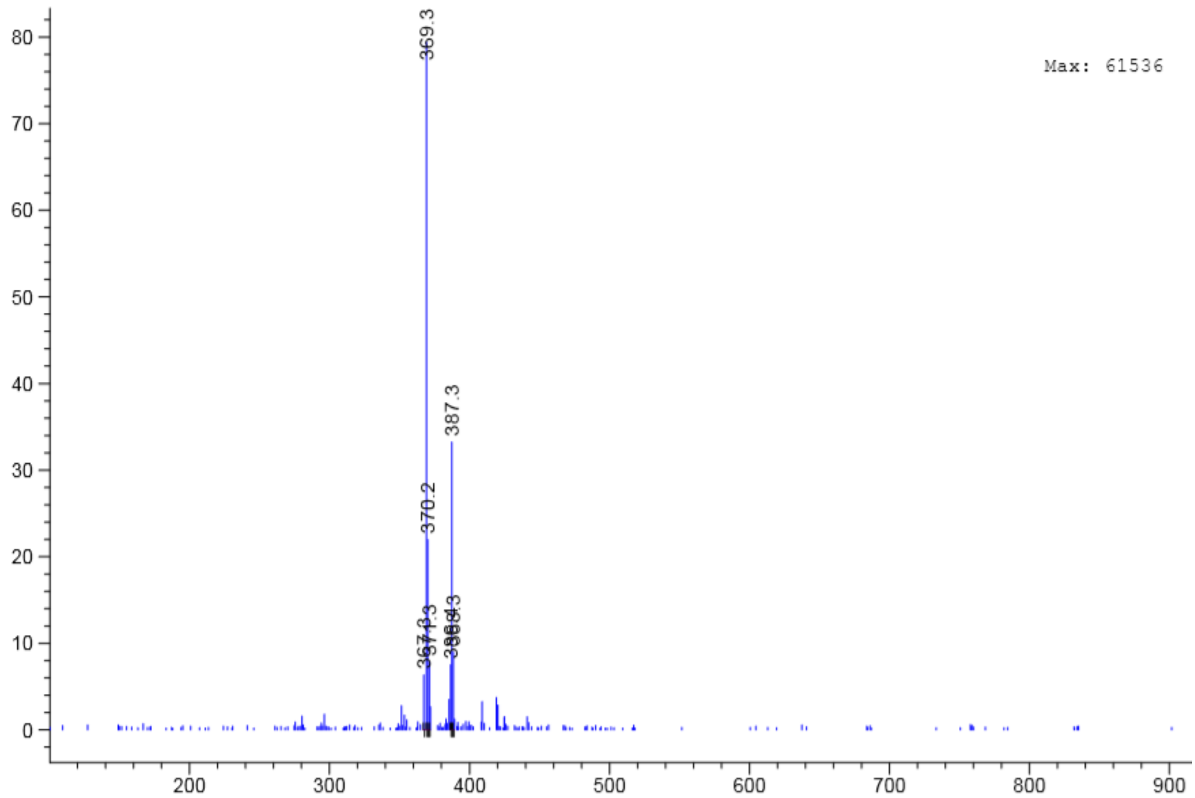

Figure S38. LRMS data of Ophiobolin C (**7**).

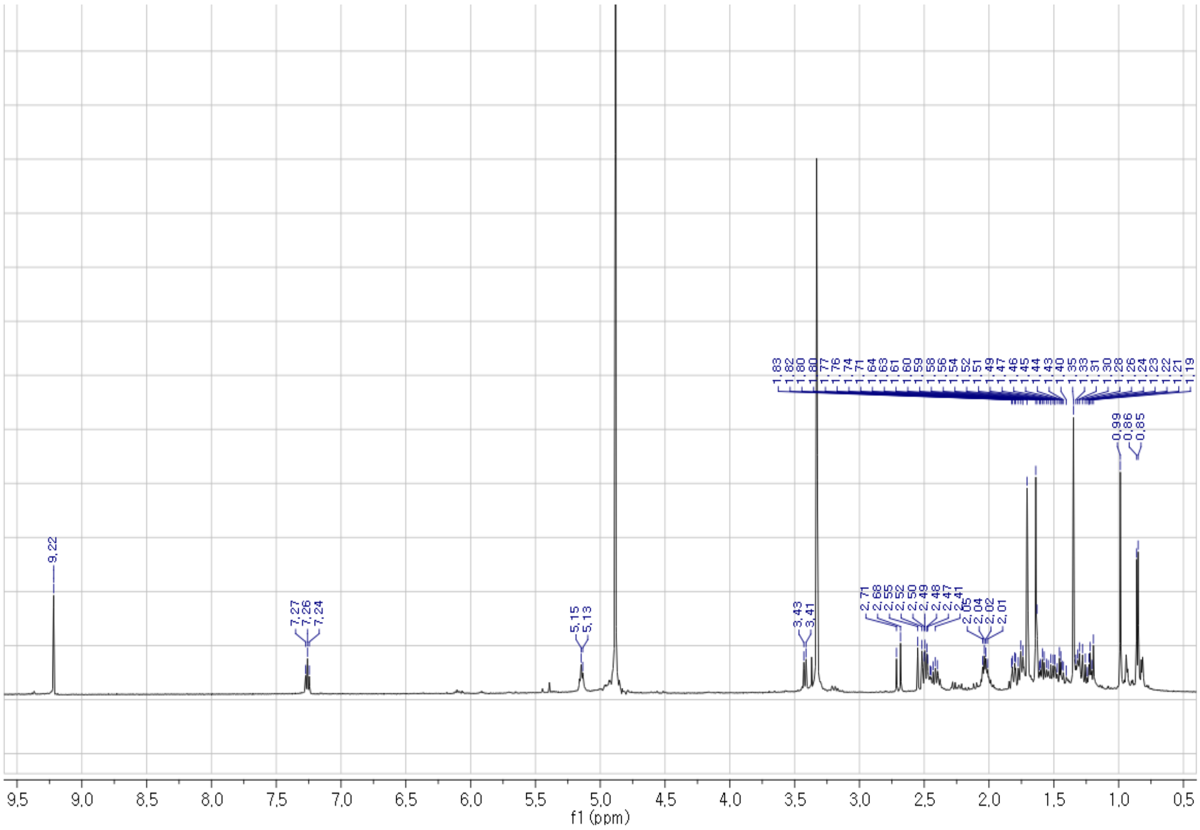

Figure S39. ^{1}H NMR spectrum of Ophiobolin C (**7**).

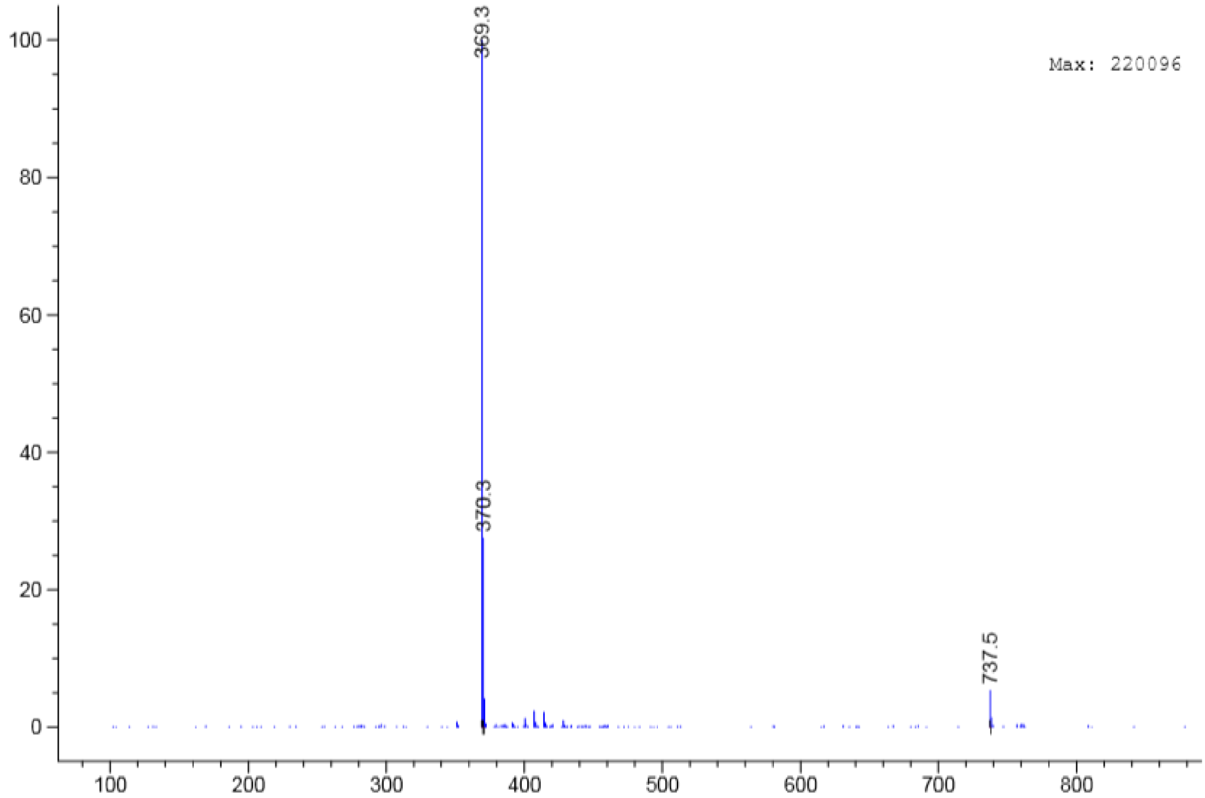

Figure S40. LRMS data of 6-*epi*-ophiobolin N (**8**).

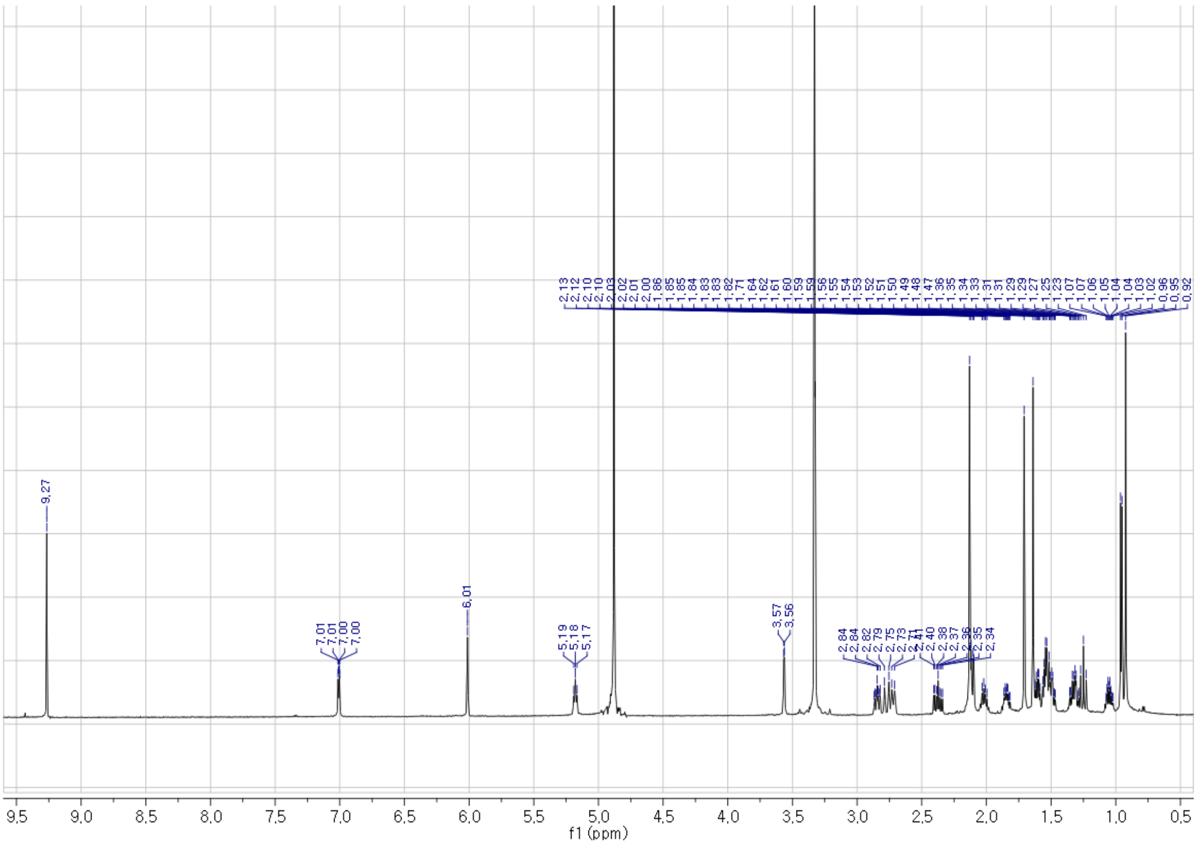

Figure S41. ^{1}H NMR spectrum of 6-*epi*-ophiobolin N (**8**).

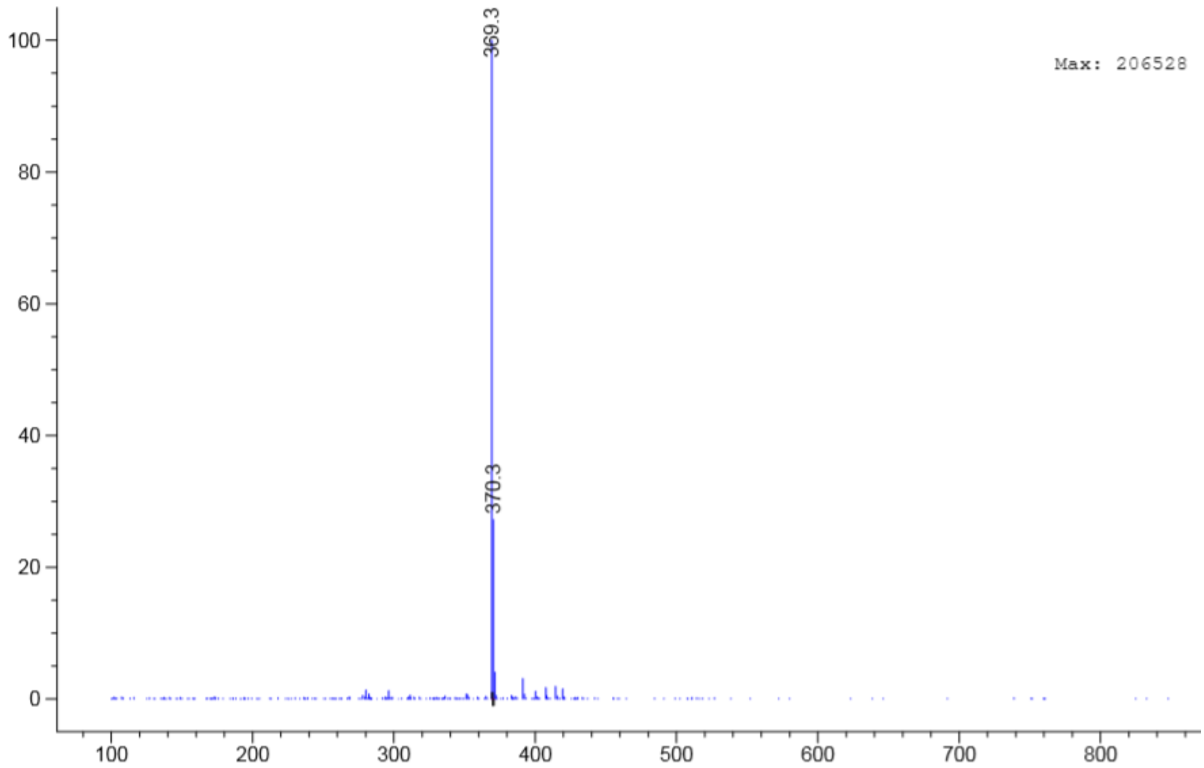

Figure S42. LRMS data of Ophiobolin N (**9**).

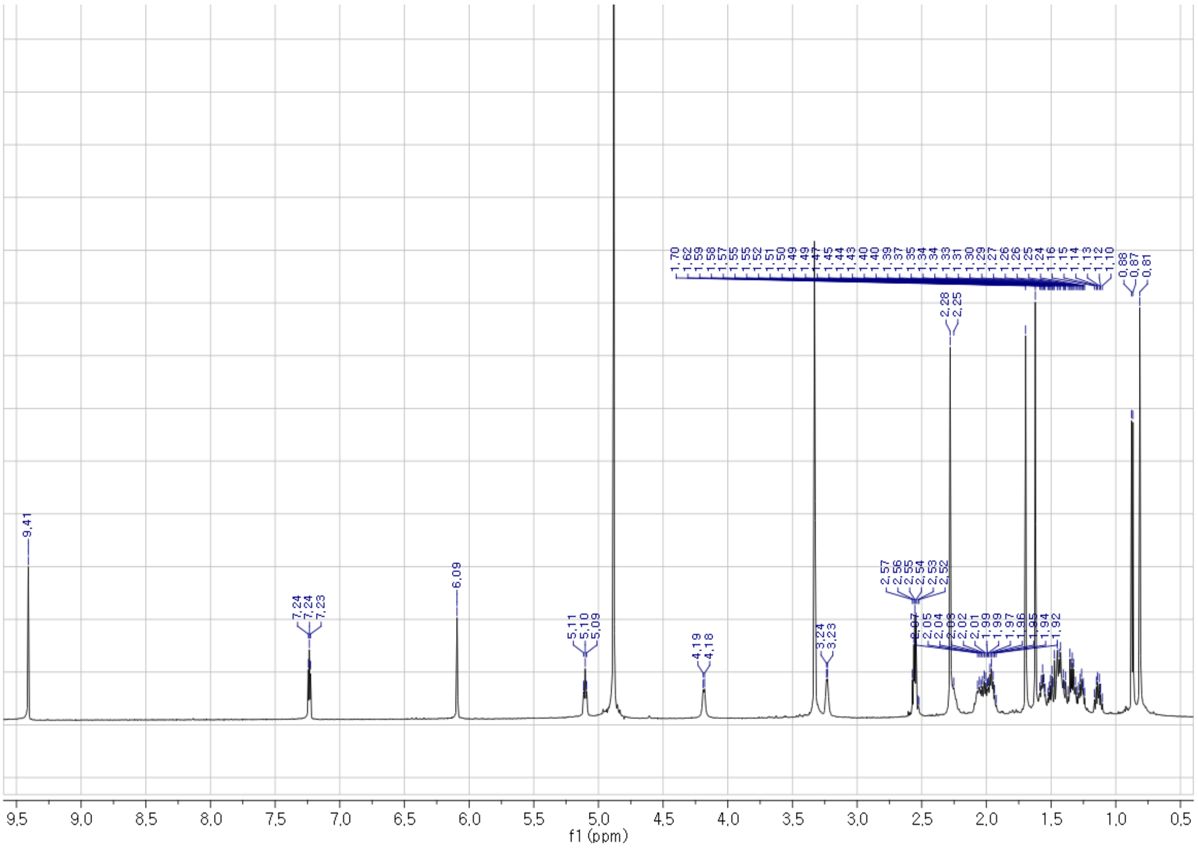

Figure S43. ^{1}H NMR spectrum of Ophiobolin N (**9**).
